# Competition in the economic crisis: Analysis of procurement auctions

**DOI:** 10.1016/j.euroecorev.2014.10.007

**Published:** 2015-01

**Authors:** Klaus Gugler, Michael Weichselbaumer, Christine Zulehner

**Affiliations:** a Vienna University of Economics and Business, Welthandelsplatz 1, A-1020 Vienna, Austria; b Goethe University Frankfurt, Faculty of Economics and Business Administration, Grüneburgplatz 1, 60323 Frankfurt am Main, Germany; c Austrian Institute of Economic Research, Arsenal, Objekt 20, 1030 Vienna, Austria; d CEPR, 77 Bastwick Street, London EC1V 3PZ, United Kingdom

**Keywords:** Construction procurement, First-price auctions, Private values, Economic crisis, Government stimulus

## Abstract

We study the effects of the recent economic crisis on firms׳ bidding behavior and markups in sealed bid auctions. Using data from Austrian construction procurements, we estimate bidders׳ construction costs within a private value auction model. We find that markups of all bids submitted decrease by 1.5 percentage points in the recent economic crisis, markups of winning bids decrease by 3.3 percentage points. We also find that without the government stimulus package this decrease would have been larger. These two pieces of evidence point to pro-cyclical markups.

## Highlights

• We study the effects of the recent economic crisis on firms׳ bidding behavior and markups in sealed bid auctions.
• Backlogs fall and competition rises in procurement auctions during economic crisis.
• Firms׳ estimated markups reduce by 1.5, winning bids by 3.3 percentage points.
• Counterfactuals show that the major part of the fall in markups comes from backlog and competition.
• Without the government stimulus package, the decrease in markups would have been larger.

## 1 Introduction

How markups move, in response to what, and why, is however nearly terra incognita for macro … [W]e are a long way from having either a clear picture or convincing theories, and this is clearly an area where research is urgently needed. Blanchard (2008, p. 18).

The recent financial crisis provides a unique testing ground for analyzing causal economic relations. Although some economists warned of the bubbly nature of asset prices (e.g. house prices, see Shiller, 2005), the financial crisis and the ensuing economic crisis were unforeseen.^1^ We use the current economic crisis to shed light on how markups move in response to an exogenous shock in demand. We investigate firms׳ competitive behavior in procurement auctions before and during the crisis; and build a bridge between micro-level behavior and the effects of macro-level shocks.

The recent economic crisis allows testing for (at least) two effects. First, it enables us to estimate the effects of the recent economic shock on fundamental structural characteristics of the economy. In particular, we test for the effects of the shock on the process and intensity of competition, i.e., pricing behavior and markups. Second, the effects of fiscal policy can be tested in an unprecedented manner. In normal times, the effects and determinants of fiscal policy are difficult to ascertain due to their endogenous nature. Activist fiscal policies in the crisis are clear cut and mostly targeted to specific industries like the construction sector. Thus, we can test for the effects of fiscal policy by constructing a proper counterfactual allowing us identification. We use data from before the economic crisis to predict what would have happened but for the state stimulus intervention.

Our theoretical reasoning and strategy of identification are as follows. In “normal” times (in the years before the recent economic crisis) firms operate at their equilibrium capacity utilization rate. Sometimes they are unconstrained, but sometimes they hit their capacity constraint. Using data from highway procurement auctions, Jofre-Bonet and Pesendorfer (2003), for example, estimate that firms are capacity constrained in 32% of the contracts during the “normal” economic time period 1996–1999, and find that bids are 18% higher if all bidders are constrained. This implies that economic rents are higher, if firms are capacity constrained. The negative demand shock resulting from the economic crisis relaxes capacity constraints: bidders have idle capacity. Given idle capacity, they participate more often in the remaining auctions and bid more aggressively since their costs are lower. In a dynamic model, if firms are capacity constrained, they may also price into their bids the lost option value of winning today versus winning later, i.e. the (additional) cost of winning today consists also of the loss of future discounted profits due to limited capacity.

Our results are based on an auction model fitted to detailed and comprehensive data from procurement auctions in the Austrian construction sector in the period 2006 to 2009. Using the start of the downturn as a quasi-experiment, the model enables us to show how firms react to the massive negative demand shock. The economic crisis has led, however, governments in many countries to implement stimulus packages and counteract these negative shocks. This has fueled interest in and discussion of the questions if, how and which government measures are effective. The Austrian government also implemented a stimulus package in response to the economic crisis. The main target was the construction sector. Applying the same logic as above, the government intervention should — ceteris paribus — increase markups.

The focus of stimulus measures on the construction industry is not idiosyncratic for Austria. Many countries implemented stimulus measures, of which the construction sector plays a large part. In Austria, the share of the stimulus packages covered by construction spending was about 22% in 2009. In Germany, about 17% of the stimulus in 2009 and 2010 were directed to construction. In the US, the share was about 13% in 2009. Thus, we believe that our results are relevant for fiscal policy in general and are not confined only to Austria.

For the empirical implementation, we follow and simplify Athey et al. (2011) and use a parametric version of Guerre et al. (2000) to recover the distribution of bidder costs from the observed bids. We assume that the demand shock exogenously changed bidders׳ participation.^2^ The distribution of bidders׳ costs is estimated based on the assumption that in equilibrium the estimates of the distribution function summarize bidders׳ beliefs and can be used to infer bidders׳ costs based on the first-order conditions of optimal auctions. Estimation of the distribution of bids is based on auction characteristics, firm characteristics as well as the level of competition to which firms are assumed to respond optimally.

We utilize a detailed data set that includes a rich set of variables. Our data set covers nearly the population of all public procurement auctions in the construction sector in Austria during the years 2006 to 2009. It includes auction specific variables such as the engineers׳ cost estimates of one firm for each project and the identities and bids of participating bidders. By matching to a firm-level database and combining it with data on travel distances from the firm׳s address to the location of the project, several bidder-specific variables are added. We are thus able to control for bidder heterogeneity in the econometric model.

We show that the crisis indeed had the expected effects on competition: the negative demand shock led to more bidders in the remaining auctions and these bid more aggressively. We find a significant decrease of the average markup in the crisis period of about 1.5 percentage points relative to a pre-crisis mean markup of 12%. The winning bids are 3.3 percentage points lower relative to 22.9% pre-crisis. Extending our analysis to the dynamic model of Jofre-Bonet and Pesendorfer (2003) shows that the main crisis effect is robust to the incorporation of the option value effect of winning. Markups increase by about 2 percentage points overall in the dynamic model. The crisis drop is about 2 percentage points for all bids and about 4 percentage points for winning bids.

We attribute the decrease in markups mainly to two characteristics that are affected simultaneously by the negative demand shock: lower backlogs of firms and an increase in the number of bidders. Because of the unforeseeable nature of the recent financial crisis, firms did not reduce their capacities to the lowered demand level. Exit did not immediately follow. Roughly the same number of firms bid for a reduced number of projects and consequently more firms bid for each project. More competition and reduced marginal costs lead to lower markups and lower prices.

A counterfactual analysis provides an estimate of the effect of the stimulus on markups. In this counterfactual, we reduce the backlogs of bidders by the amount the government spent on stimulus in the construction sector. Our estimate indicates that the drop in markups would have been about a third higher — increasing from 1.5 to 2 percentage points — without government stimulus.

While the macroeconomic literature compares markups over the business cycle across industries,^3^ we follow the microeconomic literature and estimate bidders׳ cost distribution from submitted bids in construction procurement auctions.^4^ By focusing on one industry and imposing the structure of a game-theoretic model we are able to back out firm specific cost and do not have to rely on data averaged over firms. We have to assume, however, that bidders behave according to the theoretical model.^5^

In the literature, construction procurement auctions have been extensively studied. Jofre-Bonet and Pesendorfer (2003) show non-parametric identification in repeated auction games and using a parametric model for the estimations, they find evidence of capacity constraints in Californian highway procurement auctions. Krasnokutskaya (2011) proposes a nonparametric estimation method to recover the distribution of bidders׳ private information when unobserved auction heterogeneity is present and finds that private information is estimated to account for 24% of the variation in bidders׳ costs in Michigan highway procurement auctions. Balat (2012) looks at road construction in California and investigates the effect of the American Recovery and Reinvestment Act on equilibrium prices paid by the U.S. government. He extends the models by Jofre-Bonet and Pesendorfer (2003) and Krasnokutskaya (2011) and allows for unobserved project heterogeneity and endogenous participation. His results show that prices rather than quantities increase as a consequence of the implemented stimulus. Groeger (2014) studies participation and bidding decisions in repeated highway procurement auctions and the estimated parameters suggest that participation synergies exist. Further studies accounting for endogenous participation are Krasnokutskaya and Seim (2011) and Athey et al., 2011, Athey et al., 2013.

The organization of the paper is as follows. In the next section, we describe the construction sector in Austria and provide insights into the organization of procurement auctions. Section 3 presents the theoretical background and the econometric model of the distribution of bidders׳ construction costs. We provide a discussion of the specific timing of the crisis as it hit the Austrian construction sector and describe the likely consequences of the crisis on competition. The section also contains details on the data as well as summary statistics. Section 4 presents and discusses the estimation results. Based on the estimates, we discuss the effects of the recent economic crisis and the associated fiscal stimulus on firms׳ costs and markups. In addition, we report robustness checks as well as counterfactual effects of the model, together with more detailed results of markups in the stimulus and non-stimulus sector and for specific stimulus projects. The relation to other findings and competing models as well as possible policy conclusions are discussed in Section 5. In Section 6, we conclude.

## 2 Institutional background

This section provides information on Austria׳s construction sector and how the Austrian government implemented the stimulus packages to combat the economic crisis following the breakdown of Lehman Brothers. A main part of the stimulus package concerned the construction sector and thus affected procurement auctions. Finally, we describe the organizational background of these auctions.

### 2.1 Construction sector and stimulus packages in Austria

**Fig. 1:**
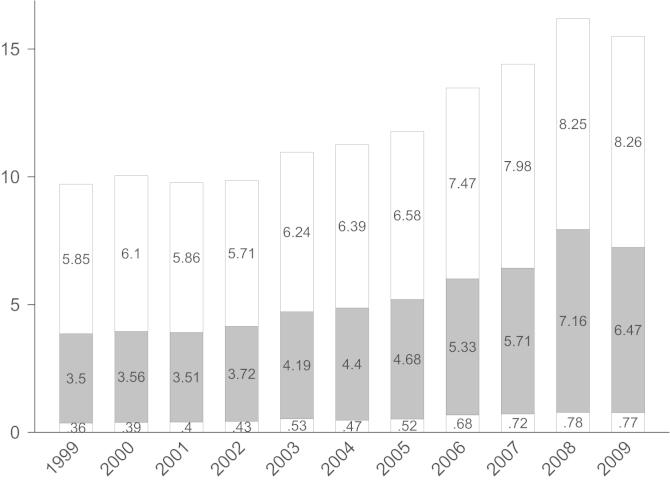
Main construction sectors in Austria, 1999–2009. *Notes*: top (white) bars building; middle (grey) bars are heavy construction and bottom (white) specialized construction activities. Values in billion Euros. *Source*: Statistik Austria.

In Austria, the construction sector accounts for 7% of GDP on average between 2006 and 2009 (in Germany this share is 4.2%, in the USA 4.4%; OECD STAN data set). As can be seen from Fig. 1, total construction value added steadily increased from around 10 billion euros in 1999 to 2002 to 16.2 billion euros in 2008 in Austria in nominal terms. In 2009, when nearly all countries went into recession due to the financial crisis, total construction value added fell to 15.5 billion euros, which is a drop of 5% in real terms (the inflation rate was 0.5%) compared to a drop in real GDP of 3.8%. Building (corresponding to 2-digit-SIC code 15) and heavy construction (SIC-2: 16) are the two main subsectors. While building construction remained nearly flat in real terms, heavy construction value added fell by around 10% in real terms.

Like other countries, the Austrian government responded to the global economic crisis and did this with similar measures and at similar dates. In October 2008, the Financial Market Stability Act and the Interbank Market Support Act became effective to support the interbank market, allow the government to grant guarantees, assume bank liabilities and acquire bank equity. Also in October 2008, the parliament voted for the first of two stimulus packages. In December 2008, the council of ministers voted for the second stimulus package.

The major parts of the stimulus packages consisted of credit and financing for private companies, a bonus depreciation plan, additional funds for R&D, subsidies for thermal rehabilitation of buildings, a car scrappage program (“cash-for-clunkers”) and increased public expenditures on infrastructure. These infrastructure expenditures were made by advancing investment on public infrastructure, originally scheduled for years later than 2009 and 2010. In 2009 — the year when our data end and the first year of stimulus — the stimulus directed at the construction sector consisted of 363.3 million euros. It was invested into roads, railways and (public) buildings. Within these categories, it was spread over all sorts of construction projects: maintenance, rehabilitation, rebuilding and new building of railway stations, roads, schools, universities, judicial buildings and so on.

### 2.2 Organization of procurement auctions

Austria׳s public authorities (federal and regional government, social security institutions, and the like) are subject to the Federal Public Procurement Law (PPL, “Bundesvergabegesetz”). Private companies have to follow these rules only if (1) they are active in a “sectorial activity” (provision of water, mail services, energy, or traffic), and (2) their activity is regulated (e.g. entry regulations). In principle, there are no lower limits for the applicability of the PPL. Upper limits are in effect, but only to enforce EU-wide announcement rules for larger projects (in construction, currently 4.845 million euros). The main purpose of the PPL is to stimulate competition and safeguard equal treatment of all potential bidders. The law stipulates some restrictions on bidders, but they do not, in our judgment, affect competition, but are intended to forestall opportunistic behavior (e.g. the requirement of a 5% bid bond). Except for very small projects the public authority must choose between an “open procedure” or a “restricted procedure with publication of a contract notice”. Such projects have to be made public and documents must be freely accessible. In our sample, 85% of the auctions are “open”, and only 2% are of the “restricted” type. The remaining 13% were not publicly announced. Instead, selected qualified companies were invited to submit bids.

All contracts are awarded by first-price sealed-bid auctions. There is no explicit reserve price – the seller has the right to withdraw the auction when the winning bid is “contra bonos mores” (offending against good morals). In court, the seller would proof this by providing a cost estimate that is based on standard commercial and professional principles and has to show that the winning bid is far higher than this estimate. Each project is described in a procurement catalogue. The seller defines the components and quantities needed for the construction project. The bidder provides a unit price for each component. The final price in the auction is then the sum of prices for the various components multiplied with the quantity. Bidders who want to participate in an auction have to proof their commercial and professional abilities before being admitted to the bidding process. On the letting day, all bids are unsealed, ranked, and the low bidder wins the auction. After the lowest bid has been identified all bidders are informed. If no bidder objects the decision within 10 days, it is final.

## 3 Bidders׳ behavior before and in the crisis

This section describes the theoretical model, the econometric model of the distribution of bidders׳ construction costs and the hypotheses for our empirical analysis. We also describe our data and give summary statistics. Our theoretical reasoning and strategy of identification are based on the assumption that in “normal” times, i.e., in the years before the Great Recession, firms operate at their equilibrium capacity utilization rate. Once firms hit their capacity constraints, their costs increase. We then expect economic rents to be higher. The negative demand shock due to the economic crisis implies that the capacity constraints are relaxed: bidders have idle capacity in the crisis. Given idle capacity they participate more often in the remaining auctions and bid more aggressively since their costs are lower.

To estimate the distribution of bidders׳ costs, we implement a static first-price auction with an exogenously given number of bidders as our main specification. As robustness checks, we also implement a dynamic first-price auction that accounts for the strategic effect of capacity constraints following Jofre-Bonet and Pesendorfer (2003), and a model with endogenous entry following Athey et al. (2011).^6^ As our main econometric model is however based on the static model, we stick to the static theoretical model in the following and provide the respective implementations of the other models in Appendix A.

### 3.1 Theoretical model

We adopt a standard first-price sealed bid auction model to describe the bidding process.^7^ We consider an auction for a single contract. The set of bidders is denoted with N =1,…,N. Bidders are assumed to be risk-neutral and their identity to be known.^8^ Bidder *i* learns her private cost for the project, *c*_*i*_, and bids in the auction. Bidder *i*׳s cost, *c*_*i*_, is an independent draw from a distribution *F*_*i*_ with continuous density *f*_*i*_ and support $[\underset{̲}{c},\overset{¯}{c}]\subset R_{+}$. Bidders independently submit bids and all bids are collected simultaneously. The contract is sold to the bidder with the lowest bid, provided that her bid is not higher than the seller׳s secret reserve price *r*, and the winner receives her bid for the contract.^9^ A bidding strategy $b_{i}=b_{i}(c_{i};N)$ specifies *i*׳s bid as a function of her cost, the number of bidders and their identity.

*Bidding equilibrium*: Bidder *i* has cost *c*_*i*_. Bidders maximize their expected profits

(1) $$\pi_{i}(c_{i},b_{i})=(b_{i}-c_{i})\underset{j\in N\backslash i}{\prod }[1-F_{j}(b_{j}^{-1}(b_{i}))],$$

where the lowest bidder wins the contract in the first-price sealed bid auction and makes a profit equal to $b_{i}-c_{i}$ and all other bidders make zero profits.^10^ The optimization yields the first-order condition,

(2) $$(b_{i}-c_{i})\underset{j\in N\backslash i}{\sum }\frac{f_{j}(b_{j}^{-1}(b_{i}))}{\left(1-F_{j}(b_{j}^{-1}(b_{i}))\right)}\frac{\partial b_{j}^{-1}(b_{i})}{\partial b_{i}}=1,$$

where we as is standard in the literature focus on Bayesian Nash equilibria in pure bidding strategies. There is no explicit solution to (2), but the first order conditions, together with the boundary conditions that $b_{i}(\overset{¯}{c};N)=\overset{¯}{c}$ for all *i* uniquely characterize optimal bidding strategies. In equilibrium, the bidders use a markup strategy and bid their values minus a shading factor that depends on the equilibrium behavior of opponents (Maskin and Riley J., 2000).^11^^,^^12^

For the estimations, it is helpful to follow Guerre et al. (2000) and rewrite bidder *i*׳s expected profit as

(3) $$\pi_{i}(c_{i};N)=\underset{b\leq r}{\max}(b_{i}-c_{i})\underset{j\in N\backslash i}{\prod }(1-G_{j}(b;N)),$$

where $G_{j}(b;N)$ $=F_{j}(b_{j}^{-1}(b;N))$ is the probability that *j* will bid less than *b* and $b_{j}^{-1}(b;N)=c_{j}$. The first order condition for *i*׳s bidding problem is then

(4) $$\frac{1}{b_{i}-c_{i}}=\underset{j\in N\backslash i}{\sum }\frac{g_{j}(b_{i};N)}{\left(1-G_{j}(b_{i};N)\right)},$$

which provides the basis for estimating bidders׳ cost distributions.

### 3.2 Estimation of bidders׳ costs

To illustrate the econometric model and the structural estimation of the distribution of bidders׳ construction costs, we use sealed-bid data to estimate the parameters of the theoretical model as a function of auction and bidder characteristics making use of the approach developed by Guerre et al. (2000). They suggest to estimate the distribution of bids in a first step and to recover the distribution of bidders׳ costs in a second step by using the first order-condition for optimal bidding behavior. With the estimates of the distribution of bidders׳ costs at hand, we then calculate markups.

Let X denote the set of auction characteristics and Z denote the set of bidder characteristics. We assume that both sets of characteristics are known to the econometrician and the bidders. Such an assumption precludes the existence of characteristics unknown to the econometrician, but known to bidders. We believe that due to the detailed data set including backlog, distance and firm size, this assumption is plausible.^13^ We model N, the set of bidder identities, with number of bidders *N* and cumulative characteristics of the other bidders that participate in the auction.^14^^,^^15^ The list of variables denoting X, Z and N is given in Table 1.

Bidders also know their private cost *c*_*i*_. We denote the distribution of bidders׳ costs as $F_{i}(\cdot |X,Z)$, and assume that bidders׳ costs are independent conditional on (X,Z). Given these assumptions, one can write the distribution of bids as $G_{i}(\cdot |X,Z,N)$.

*Distribution of bids*: The first step of Guerre et al.׳s (2000) approach to obtain an estimate for the distribution of bidders׳ cost is to estimate the distribution of bids. Their approach is very general and allows the non-parametric identification and estimation of the distribution of bidders׳ cost. Here, we adopt a parametric approach. Conditional on the observable auction and firm characteristics (X,Z), and the set of bidders identities N, the joint distribution of bids in a given auction is the distribution $G_{i}(\cdot |X,Z,N)$. We specify the Weibull distribution as the distribution of bids:

(5) $$G_{i}(b_{i}|X,Z,N)=1-\exp\{-{\left(\frac{b_{i}}{\lambda_{i}(X,Z,N)}\right)}^{\rho_{i}(X,Z,N)}\},$$

where $\lambda_{i}(X,Z,N)$ is the scale and $\rho_{i}(X,Z,N)$ is the shape of the Weibull distribution. We parameterize the scale as $\lambda_{i}(X,Z,N)=\lambda_{0}+\lambda_{X}X+\lambda_{Z}Z+\lambda_{N}N$ and the shape as $\rho_{i}(X,Z,N)=\rho_{0}+\rho_{X}X+\rho_{Z}Z+\rho_{N}N$. We estimate the parameters of the model, (λ,ρ), by maximum likelihood.

*Distribution of costs*: Assuming that bidders behave as predicted by the model, the distribution $F_{i}(\cdot |X,Z)$ is identified from the distribution of observed bids. The advantage of this approach is that no differential equation has to be solved and no numerical integration has to be applied. The estimation of bidders׳ costs is directly derived from identification and can be expressed as

(6) c^i=ϕi(bi;X,Z,N)=bi−1∑j∈N\ig^j(bi|X,Z,N)1−G^j(bi|X,Z,N),

where G^j and g^j are estimates of the distribution and the density of bidder *j*׳s costs, respectively. Bidder *i*׳s estimated cost are a function of her equilibrium bid and the joint distribution of her rivals׳ equilibrium bids. If we observe all bids and bidder identities, then the asymmetric independent private values model is identified (Campo et al., 2003, Laffont and Vuong, 1996, Li and Zhang, 2014) and it is then straightforward to construct an estimate of *ϕ*_*i*_ given the estimates of *G*_*i*_ and *g*_*i*_, i.e. G^i and g^i.^16^^,^^17^^,^^18^ With the pseudo sample of bidders׳ costs, c^i, and provided that *ϕ* is invertible, we are able to calculate the distribution of bidders׳ costs as

(7) Fi^(c^|X,Z)=Gi^(ϕi−1(c^i;X,Z,N)|X,Z,N).

Finally, we assume a unique equilibrium or that the selected equilibrium is the same across observations. If this is not case, we would not match the distribution characterizing a bidder׳s beliefs in a given auction, as the observed distribution of opponent bids would be a mixture of those in each equilibrium.

### 3.3 Timing of the crisis and empirical hypotheses

In our empirical analysis we want to exploit the economic crisis as a quasi-natural experiment after which firms in the construction sector start going into public procurement auctions more often. It is not obvious when the demand changes were perceived as signs of an economic crisis and consequently when firms changed their behavior. Nevertheless, we want to identify a rough date when to expect changes borne by the factors outlined above.

**Fig. 2:**
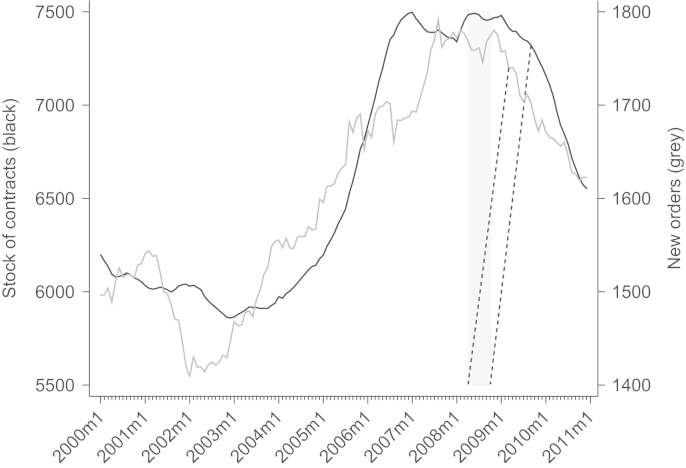
Stock of contracts and new orders in construction indicate when crisis started. *Notes*: shaded area potential start of the crisis derived from stock/new orders. Values in million 2005 Euros. *Source*: Statistik Austria, Wifo database.

Fig. 2 puts developments in the construction sector in a longer time perspective. Total construction (private and public contracts) displays an upward trend both in the stock of contracts and new orders — new orders are the gross inflow of new contracts that are accepted by firms in a given month — up to about 2007. Stocks of contracts reach a plateau a few months earlier than new orders. Not before September 2009 do the stock values fall short of the level seen in January 2008. Subtracting eleven months leads to the first possible impact of the trailing moving average. October 2008 then is a potential date when we could expect changes in the bidding behavior of firms if stock values of contracts are relevant for the bidders. New orders provide early information on the output of the construction sector in the months that follow. Construction firms can anticipate their backlog in the near future and may adjust bidding behavior. A relatively consistent downward movement of new orders starts in March 2009, which, after subtracting eleven months, gives April 2008 as a second relevant date.

**Fig. 3:**
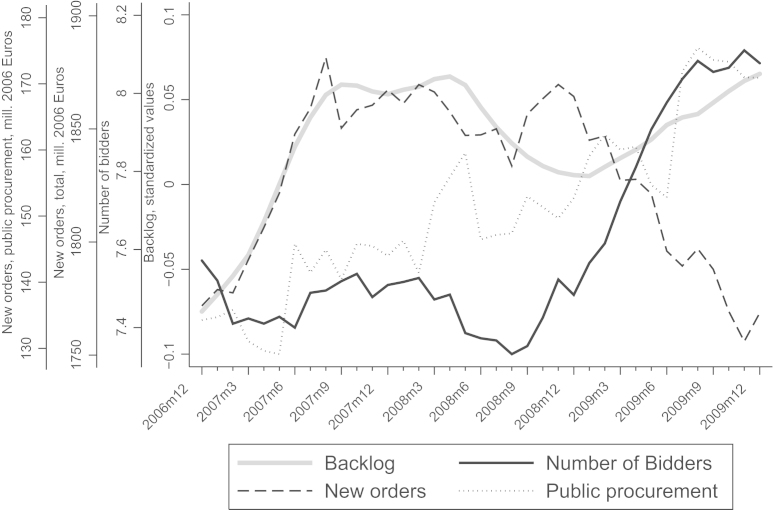
Development of the average number of bidders, construction activity and backlog. *Notes*: 12-period trailing moving averages; backlog is calculated as in Jofre-Bonet and Pesendorfer (2003) based on the estimation sample, as is the number of firms; flow of orders in construction sector and in public procurement from Statistik Austria.

In the crisis demand shrinks, capacity constraints are relaxed, and (roughly) the same number of bidders bid in fewer auctions, implying an increase in the number of bidders per auction. Thus, due to this competition effect, bidders bid more closely to their true costs in the crisis. Fig. 3 shows a striking negative relationship between the number of bidders per auction and the new orders series in the construction sector (both public and private contracts).^19^ While new orders in the construction sector sharply decrease due to the crisis, the number of bidders per auction sharply increases. New orders in public procurement (also in Fig. 3), however, are larger in the last few months of 2009. Accordingly, our backlog measure — after dropping sharply in 2008 — begins to rise again in early 2009.^20^ We attribute this to the growing inflow of stimulus projects.

To provide further evidence for our argumentation that the crisis affected the number of bidders in public procurement auctions and bidders׳ capacity utilization measured by their backlog, we show descriptive regressions in Table 2. We regress each variable on a dummy variable that is zero before and one in the crisis — starting in October 2008 — and a time trend to control for general movements in the time series.

The results depicted in Panel A of Table 2 confirm our impressions from Fig. 3. We observe that the number of bidders in an auction increases in reaction to the crisis. This effect is significantly different from zero and about one and a third bidder on average. In columns (2)–(5), we present the robustness of our estimate to placebo treatments. One might be concerned that the increase in the number of bidders picks up some additional unspecified effects over time. Similar to Black et al. (2008), we introduce a placebo treatment and add hypothetical crisis dates to the specification. We use four additional dates for the crisis: two months and six months before and in the crisis. The estimated coefficient for the change in the number of bidders does not change significantly.

We repeat this exercise for the backlog. We find that the estimated effect of the crisis on the backlog is negative as expected and significantly different from zero. Again we run placebo experiments and find that the estimated coefficient does not vary much over the specifications shown in columns (2)–(5) in Panel B of Table 2.

In the presence of a negative demand shock, the incentive to shade bids above one׳s cost decreases. A bid is equal to the expected cost of the competitors conditional on the competitors׳ costs being less than the bid. If there is more competition in the sense that there are more actual and capacity unconstrained bidders in an auction, the markup goes down, (1) since the expected conditional cost of competitors decreases, so bids are closer to costs, and (2) capacity unconstrained bidders bid more aggressively since their costs are lower. This implies that bidders׳ expected rents go down in equilibrium and gives the first hypothesis.

*Hypothesis* 1: The markup goes down in an economic crisis.

As argued, our structural approach allows us to measure the impact of activist fiscal policy. The state intervened drastically during the crisis with the two stimulus packages, and particularly so in the construction sector. Economically, targeted fiscal stimulus shifts the demand curve in the construction sector at least partially back to where it would have been without the crisis. This gives our second hypothesis.

*Hypothesis* 2: The markup would have decreased by more had the state not intervened in the construction sector.

If validated, both hypotheses imply pro-cyclical markups. The main underlying assumption of our two hypotheses is that an increase in the number of bidders yields lower procurement prices and lower bidder revenues. This is a standard competition argument, but need not always be true. In a model with common values,^21^ informational asymmetries such as the “winners׳ curse” may offset the above argument. We argue that the independent private value model is valid in our context. As described in Section 2.2, construction projects consist not only of one (final) price, but are actually the sum of prices for various components expressed in fixed quantities that are known from the procurement catalogue. Firms put in the prices they are willing to offer for the various components. As the components of the project are fixed,^22^ firms then differ only by their private cost. However, even in an independent private values model as we apply here, bidder asymmetries^23^ or costly bidding^24^ may also lead to the effect that more bidders could lead to reduced price competition. In our case, the empirical model allows for bidder asymmetries as we control for firm specific variables such as the distance between firm location and construction site as well as firm size, and estimate bidder specific distributions. In addition, we run a robustness check modelling participation. Finally, we do not consider bidders to be risk averse, but risk neutral. If, however, bidders׳ attitude towards risk would change in the crisis, we may attribute some of the change in markups incorrectly to competition as risk aversion induces more aggressive bidding than risk neutrality (Maskin and Riley, 1984).^25^ While estimating a model with risk aversion would go beyond this paper, we can at least partially account for changes in risk aversion over time by splitting the sample and estimating two different cost functions as we do as one of our robustness checks.^26^

### 3.4 Data and summary statistics

One large Austrian industrial construction company provided our main data, containing bids, firm names and auction characteristics. This data set covers all auctions in both building and heavy construction in the period January 2006–December 2009, where this company took part either as the parent company itself or as one of its subsidiaries. According to the company, the database covers more than 80% of all auctions which must be conducted according to the Public Procurement Law. Thus our sample covers nearly the population of all public procurement auctions in Austria during the four years 2006–2009. Within this period, our database reflects on average 14% of Austria׳s total construction sector. In 2009, this share is at 16%, which mirrors the increased activity of the state through the stimulus. This increase is more visible in heavy construction, where our data set represents 26% of total heavy construction in 2009, up from an average of 21%. Our data set covers a relatively stable share of 10% of building construction.

Table 3 describes our sample selection constraints. Not all auctions in our data set go into the estimations due to missing values, mainly because of the absence of an engineer estimate in 43% of auctions.^27^ Some auctions have engineer estimates that are far off all the bids finally submitted. Because of the expected strength of the engineer estimate in determining the overall level of the bids submitted, we worry that an unexplainable large difference between the bids submitted in a project and its engineer estimate might put too much weight on auctions where it is unclear what is going on. 180 auctions fall under our definition of “outliers”.^28^ Several firm-specific variables enter our auction model. To obtain these data, we manually matched the bidding firms to Bureau van Dyck׳s Amadeus database, which contains nearly the population of companies in Austria. Matches were primarily based on company names. The firm׳s postal code was mostly available as additional information. Almost all bids were made by firms that are well known in Austria and could be matched based on their name without concern. To avoid confusion of firms with equal names, all matches have been complemented by extensive internet search. Eventually, in our main econometric model, 1655 different firms are identified taking part in 2067 auctions.

Of these firms, 1342 or 81%, were matched to Amadeus. Thus, 96% of the total 29,850 bids come from matched firms. The population of Austrian construction firms, as reported by the Austrian federal agency, Statistics Austria, consists of 4796 firms on average for 2006–2009 (building: 3958, heavy: 838). Therefore, about one-third of all construction firms submitted bids in the procurement auctions.

Table 4 gives detailed yearly summary statistics on firms, auctions, bids and competitive structure.

*Bidder characteristics*: We cover many relatively small firms with an average of around 150 employees (median: 54 employees) and 20 million euros in total assets (median: 4 million).

*Auction characteristics*: Splitting the sample into the two main construction sectors reveals that two-thirds belong to building and the rest to heavy construction. On average, the technical estimate for the projects׳ cost is 2.4 million euros (at 2006 constant prices) and the average winning bid is 4% below that. Transportation costs play an important role in the construction sector. We measure these costs by the distance of a firm to a construction site. Based on the construction sites׳ and bidders׳ postal codes, we used Microsoft׳s Bing Maps to calculate the driving distances for all bidders to the project sites corresponding to the auctions. Postal codes for virtually all bids are available in our main data. 4-digit postal codes give very detailed information on location in Austria and exact street addresses can be neglected without loss of relevant information for our purpose.^29^ Besides driving distance, we have also retrieved travel times. While in singular cases the two may differ substantially, the correlation is very high at 0.973 in our sample. Not surprisingly, all calculations are robust to the choice between them or the inclusion of both. Mean distances equal 123 km in 2006 and 2007, reach its highest value of 139 km in 2008 and fall slightly to 135 in 2009. Thus, firms are willing to bid in projects farer away and thus to drive longer distances to the project sites in the crisis.^30^

*Competition/set of bidder identities*: To model competition within auctions, we have to approximate the set of bidder identities N. We use the actually participating bidders and cumulative characteristics of other participating firms such as the sum of distances or the sum of backlogs. In the years 2006–2008, there are on average around 7.5 actual bidders. In 2009, during the crisis, this number goes up to 8 bidders. We also use the number of potential bidders. Several conditions are imposed on the firms in the sample to qualify as a potential bidder. First, only firms that submit a bid for any heavy construction auction in the sample are permitted as potential bidders for any other heavy construction auction; the equivalent holds for building construction. Second, the auction must be smaller than three times the maximum value of projects a firm has ever bid for in the sample. Third, a firm is allowed as a potential bidder only if the auction is within the maximum distance that it ever bids in the sample. Finally, the auction must be within a district where the firm has bid in the same calendar year. Each step of these cumulative restrictions has been used to arrive at alternative, larger numbers of potential bidders. The main results were not affected by alternative definitions of potential bidders. Table 4 summarizes the results for two definitions: the most restrictive one, where all conditions hold (“def. 1”); and a less restrictive definition, where only the first two conditions are applied (“def. 2”). On average, 30 firms qualify as potential bidders per auction under definition 1, ranging from 2 to 83, and 663 potential bidders qualify under definition 2. We show the regressions with definition 1. They are robust to a change in the definition of potential bidders.

*Capacities*: Backlog used by a firm at a point in time is calculated as in Jofre-Bonet and Pesendorfer (2003). Every project is added to the backlog when a firm wins it. Projects are linearly worked off over their construction period, which releases capacity. Every firm׳s backlog obtained is standardized by subtracting its mean and dividing by its standard deviation. We have also experimented with alternative definitions of backlog but they do not change our findings substantively. The variable captures all auctions of the company providing us with the data but does not carry information on backlogs of auctions where this firm did not participate. Although this company participates in most auctions, potential bias could be introduced. To relieve the variable from potential bias, additional data about projects won in the past come from the “Austrian Register of Tenderers”. The register gathers all public procurement tender offers and information about the winners and often the contract value of past concluded contracts. We selected those projects where at least one recorded subclassification belongs to the construction sector (Common Procurement Vocabulary 2-digit code equal to 45). By matching the winning bidder and thus the winning bid, where available, to the main data set, unbiased procurement backlog data are available for all companies. A backlog variable needs some precursory data to account for projects already in the books of the firms when our main data set starts. The additional information from these external data contains projects before 2006. We go back until 2005, one year before our estimation sample starts, in our matching. Of the 5235 winning bids in the Register, 42% come from firms that also bid in our main data. These winning bids correspond to 72.3% of all project values won in the Register. Keeping in mind that some of the projects from the Register that we retrieved are primarily other-than-construction projects and have often only one subclassification from the construction sector, this high percentage of procurement values from matched firms gives further indication that our main data is representative. To proxy firms׳ outside option, we use the inflow of new orders into the construction sector, i.e., a macro variable, in our regressions. Herewith, we can account — at least to some extent — for contracts that have not been procured.

*Stimulus sector and stimulus projects*: Stimulus in the construction sector took place predominantly in three incorporated but government owned institutions for managing public buildings (Bundesimmobiliengesellschaft), public highways (Autobahnen- und Schnellstrassen-Finanzierungs-Aktiengesellschaft) and public railways (Österreichische Bundesbahnen). From public information we could identify which specific projects in the sample were designated by the government for the stimulus package. 17 of the 561 projects in the estimation sample in 2009 are part of the stimulus package, representing 3.8% of the total project value of 2009. These specific projects were scheduled after the year 2009 originally, but were actively brought forward by the government to counteract the crisis. We thus differentiate between two types of stimulus by the government. First, the government did not abandon projects as the private sector did. We call this sector “stimulus sector”, and it includes essentially the projects of the aforementioned three institutions (and it includes also the 17 specific stimulus projects). Second, we separately look at these 17 additional projects and call them “stimulus projects”.

*Relation between engineer estimate and bids ranked first and second*: Table 5 shows the absolute values of the engineer estimate and the winning bid in columns (1) and (2), tabulated by the number of bidders. As the number of bidders increases, the relative difference between winning bid and engineer estimate falls (column (3)). Column (4) reports the number of auctions for each number of bids submitted in the estimation sample. In the lower part of Table 5, “money left on the table”, the relative difference of second-ranked to winning bid, which is a measure of informational asymmetries, is tabulated against the number of bidders. This relative difference is on average 7.7% and it narrows as the number of bidders increases. These statistics are consistent with the findings of Jofre-Bonet and Pesendorfer (2003), Balat (2012) and Krasnokutskaya (2011).

Columns (6)–(8) divide the auctions into projects not in the stimulus sector, in the stimulus sector and the 17 specific stimulus projects we could identify. “Money left on the table” appears to be larger for stimulus related projects.

## 4 Effect of the crisis on firms׳ markups

In this section, we describe the estimation results. Based on these results, we calculate bidders׳ markups. We exploit the recent economic crisis as a quasi-experiment and test whether firms׳ pricing power is affected by the recent economic crisis and the stimulus. We then check the results for robustness. Finally, we run a counterfactual analysis to validate the results from the quasi-experiment and test our empirical hypotheses.

### 4.1 Estimation results

Columns (1) and (2) of Table 6 show the estimates for the scale parameter *λ*, columns (3) and (4) for the shape parameter *ρ*. Backlog (i.e. own backlog) and backlog sum (i.e. the backlog of the other actual bidders) measures capacity constraints. As expected, both backlog measures display positive coefficients. Firms bid higher if their own backlog is higher since they have higher opportunity costs; and firms strategically bid higher when they know that their competitors have higher backlogs, and therefore higher costs. The effect of the log of the number of bids is negative — consistent with the notion that more bidders increase competitive intensity. Note that the log is used to account for the diminishing influence of an additional bid as the number of bidders grows.

The engineer estimate is the most important determinant of the bid. The log of the number of employees, our firm specific size measure, has a positive influence on the bid level. KM measures the driving distance between firm and project site in kilometers. While insignificant, two other variables derived from the distance data are significant: KM sum, defined as the sum of competitors׳ distances, and KM average, the average distance of the bidders in the auction. We interpret these as strategic variables in a limit pricing context: the further competitors are away from the project site, the higher their costs. When competitors are further away, a firm can submit higher bids. Besides that, some control variables are included: same postal code of firm and project site, same district and same state, open format auctions, heavy construction auctions, and bidder is a general contractor.

**Fig. 4:**
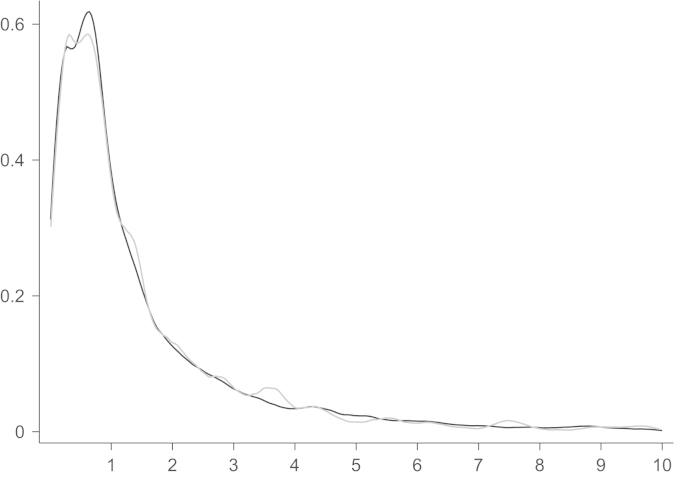
Kernel density estimate of bids and predicted bids. *Notes*: horizontal scale in million 2006 Euros. Black line represents actual bids, grey predictions. Largest 3.92% truncated.

**Fig. 5:**
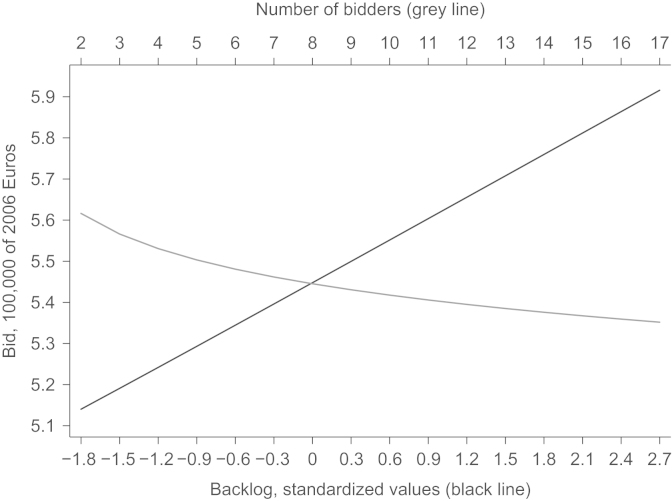
Effect of backlog and the number of bidders on the bid distribution. *Notes*: calculations based on the median project and estimated parameters of the bid distribution. The ranges of number of bidders and backlog include 99% of the observed covariates׳ variations. Bids in 100,000 Euro as of 2006. Effect of backlog (black line); effect of number of bidders (grey line).

Fig. 4 compares the kernel densities of actual to predicted bids. The prediction matches the actual density closely. To assess our two main effects more closely, we show the effect of the backlog and the number of firms on bids in Fig. 5. For the illustration we chose the median project. The ranges of backlog and the number of bidders are chosen such that 99% of all bids are covered. Both variables have the expected sign in the estimation of the scale parameter. In general, the shape parameter also influences the expected value and the total effect of a variable in the Weibull distribution is not obvious from the estimates. For our results, the partial effect of backlog or number of bidders turns out as hypothesized, too: more bidders in the auction decrease the bid, a higher backlog increases the bid.

### 4.2 Quasi-experiment

**Fig. 6:**
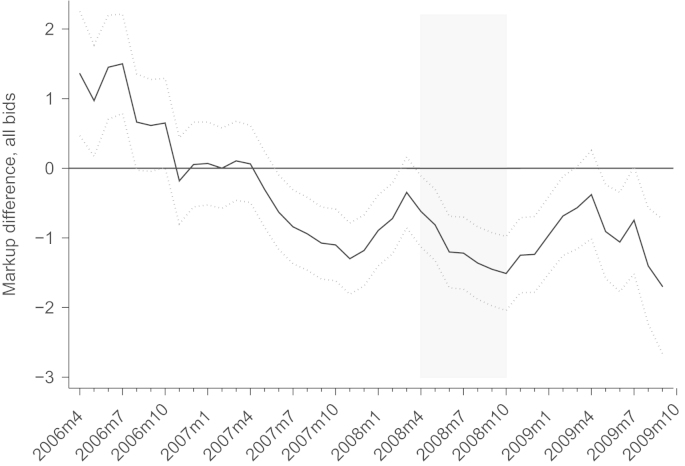
Difference between crisis and pre-crisis sample split, all bids. *Notes*: difference of mean markups before and after a specific month (black line). Dotted lines mark the upper and lower limit of the 95% confidence interval of a *t*-test. Shaded area potential start of the crises.

From our structural estimates we proceed with the calculation of the markup for each bid.^31^ With the calculated markups, we test the first hypothesis on the effects of the economic crisis on markups, i.e., that markups go down in an economic crisis. To obtain our results, we are interested in the mean difference of markups between before the crisis started and afterwards. As has been shown in Fig. 2, new orders and stock of contracts start downward developments in April 2008 and October 2008. If we split the sample in April 2008, the difference between before April 2008 and after April 2008 in mean markups is −0.6 percentage points; if we split the sample in October 2008, the difference reaches significant −1.5 percentage points. The shaded area in Fig. 6 displays these differences on the black line at the left border of the shaded area (April 2008) and right border of the shaded area (October 2008). We further show those values when we split the sample at any arbitrary point in time between April 2006 and October 2009. Note that the sample size is constant at *n*=14,845, but the subsample of the first part — the pre-crisis period — shrinks when we move the crisis start date to earlier months and the subsample of the second part — the crisis period — grows accordingly. The black line gives the difference of mean markups before and after a specific month. The dotted lines above and below the black line show the upper and lower limit of the 95% confidence interval and indicate whether the difference is significant. Reassuringly, the graph of markup differences displays a downward trend indicating that the crisis truly reduced markups.

**Fig. 7:**
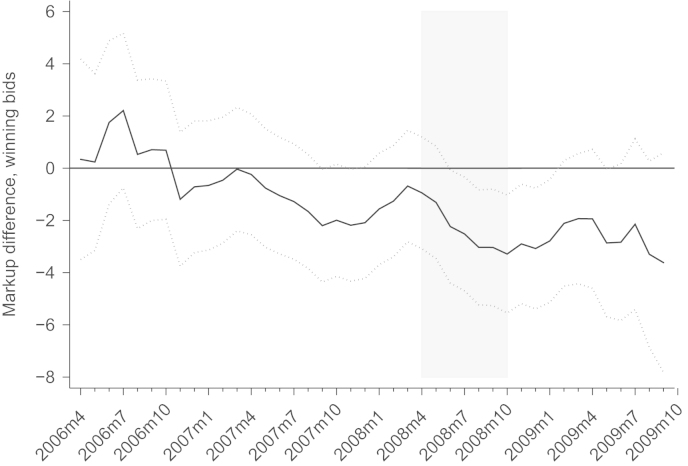
Difference between crisis and pre-crisis sample split, winning bids. *Notes*: Difference of mean markups before and after a specific month (black line). Dotted lines mark the upper and lower limit of the 95% confidence interval of a *t*-test. Shaded area potential start of the crises.

The actual prices paid are determined by the winning bids. Fig. 7 shows the mean markup differences when only winning bids remain in the calculations. The point estimate of the mean difference between before April 2008 and after April 2008 is −0.9 percentage points and therefore larger than for all bids at the same date, but not significant. The difference becomes −3.3 percentage points if October 2008 is chosen as the crisis starting date, and this number is both economically large and statistically significant. For the (eventually relevant) winning bids, therefore, the reduction of markups due to the crisis is more pronounced.

*Stimulus sector and stimulus projects*: In Table 7, we distinguish between all projects, projects in the stimulus sector and stimulus projects. Distinguishing the markups for these subgroups is of descriptive interest. We observe that markups in the stimulus sector are higher than average markups. This is true before and in the crisis. We also observe that the drop in markups is more pronounced in the stimulus sector than on average. Markups of stimulus projects are larger than markups in the stimulus sector, when we consider all bids.

Summarizing, from our structural estimates we conclude that markups decrease in the economic crisis and confirm Hypothesis 1.

### 4.3 Threats to identification

The identification of our results strongly depends on the assumption that the crisis is a quasi-experiment that exogenously decreased the demand for construction projects. We argue that there was a sudden downturn in the construction sector caused by the crisis and essentially the same number of firms is competing for fewer projects. As a consequence we observe a higher number of bidders per project, more competition in the procurement auctions and, therefore, lower equilibrium prices and lower markups. The advantage of our empirical setup is the clear time-line and its simplicity. The crisis is an exogenous demand shock, and we restrict our attention to a simple static framework.

There are, though, threats to our identification. One could think that not only demand was affected, but also bidders׳ cost. In the crisis, credit access and interest rates as well as input prices may have changed. One other source of concern might be the effect of the engineer estimate. Since one firm provided us with the data, it could be biased towards any idiosyncrasy of that firm and yield biased estimates of bidders׳ costs. It is also conceivable that the largest firms behave differently than the fringe firms. In addition, we do not account for unobserved auction specific heterogeneity and endogenous entry. Finally, as we do not consider the dynamic strategic effect of backlogs, we may misinterpret our results due to an omitted variable bias.

In order to confront these problems we provide additional pieces of evidence that should lend credibility to the interpretation of our results as market responses to the exogenous demand shock. First, we investigate whether bidders׳ cost distributions have changed in the crisis. We split the sample into the period before and during the crisis and estimate bidders׳ cost distributions separately for these two periods. Second, we address the engineer estimate and look at several modifications of our sample. Third, we include fixed effects for the seven largest firms.^32^ Fourth, we allow for unobserved heterogeneity, endogenous entry or dynamic considerations. When we model bidders׳ costs, we assume that the set of auction characteristics is known to the econometrician and to the bidders. To relax this assumption, we follow Krasnokutskaya (2011) and allow for unobserved heterogeneity. Neglecting entry cost or the dynamic strategic effect may bias our results. Thus, we estimate a model with endogenous entry similarly to Athey et al. (2011) and a dynamic model following Jofre-Bonet and Pesendorfer (2003).^33^

Our first robustness check allows the cost distribution to differ across periods. Panel A of Table 8 presents the estimated coefficients of the cost distribution before the crisis and Panel B during the crisis. We observe that, for example, the estimated coefficients for the number of bidders and for the backlog differ. The effect of the number of bidders becomes stronger in the crisis, whereas the effect of the backlog becomes weaker. This reinforces our main argument how the crisis affected this market: competition increases and capacity constraints relax. We then calculate the markups based on these cost distributions and present them in Table 9. Column 1 contains the results from the base model for comparison. The second column, R1, shows the estimated markups when we allow the cost distribution to differ over the two periods. Our main result of lower markups in the crisis holds. Interestingly, the difference is slightly more pronounced on average. This is true for all bids and for the winning bids.

The next three robustness checks in Table 9 address the engineer estimate in one way or the other.^34^ For column R2, the bids of the firm supplying the engineer estimate are omitted from the estimation of the determinants of bids. Results are robust to this modification. In R3, the engineer estimate variable is dropped altogether, nearly doubling the sample size. Although the crisis drop in markups is still present, the magnitude of markups is much larger than in the base estimate. Clearly, one has to control for auction size. For the next check, R4, also auctions with very poor engineer estimates, in terms of large deviations from the bids that were submitted eventually, were included. Consequently, the sample size is a bit larger than that of the main results in Table 6. While average markups increase, the pre-crisis–crisis pattern remains.

R5 shows the results when fixed effects for the largest seven firms in terms of total number of bids submitted in the sample are included. The fixed effects permit a somewhat different behavior by the largest firms, similar to the split into main bidders and fringe firms found in Jofre-Bonet and Pesendorfer (2003) or Athey et al. (2011). Results are robust to the inclusion of firm fixed effects.^35^ In the next column, R6, the model switches to a Weibull-Gamma-mixture distribution to permit unobserved auction heterogeneity similar to Krasnokutskaya and Seim (2011) and also Athey et al. (2011). Average markups go down, but again our main result of decreased markups in the crisis holds up. In column R7, we account for entry. The results are very close to the markups calculated in the base model. Winning bids׳ markups are slightly larger after accounting for entry. Looking at the markup difference pre-crisis versus crisis, both sets of simulated markups yield results close to the base model. In column R8, we depict the results from the dynamic model. Again, our main result prevails, although — as expected — estimated markups are higher when we account for the dynamic strategic effect.

Overall, our results are robust to a number of modifications of our main specification.

### 4.4 Counterfactual analysis

Three counterfactual experiments help to validate the results: two for the quasi-experiment and one to give a quantitative measure for our second hypothesis, i.e., the markup would have decreased by more had the state not intervened in the construction sector. To model the crisis, we manipulate the number of bidders and the backlog (own backlog, other bidders׳ backlog and new contracts). We conduct the analysis for the base model and the dynamic model. The split date for the pre-crisis vs. crisis period is the start of October 2008.

For each experiment, we recalculate the bids and the backed out cost valuations. Bids are predicted using the Weibull model (5) from the base specification. To obtain predictions for bidders׳ cost, we estimate a Weibull model using as covariates variables that influence a bidder׳s cost such as own backlog, new contracts, distance, number of employees and the engineer estimate.^36^ For the counterfactual analysis, we manipulate own backlog, backlog of others, new contracts and the number of bidders in the bid distribution and own backlog and new contracts in the cost distribution.

In the first experiment, we address the following question: what are the markups if the competitive situation did not change in the crisis? We apply the pre-crisis competitive environment in terms of number of bidders and bidders׳ backlogs to projects in the crisis. We define this environment as one bidder less and a backlog that is increased by one standard deviation. We also increase the value of new contracts by 5% to account for bidders׳ outside option in the private sector. According to the model, fewer bidders and higher backlogs will then lead to higher markups.

Table 10 shows the implications of the base model and the dynamic model for all bids and for the winning bids. For both, all bids and winning bids, we first show the mean markups for the base model and then for the dynamic model. The results from our first experiment show that we can replicate our quasi-experimental results very well on average. Depicted in column (1), we predict average markups of 12.53% for projects procured in the crisis but evaluated in a pre-crisis environment (+1bl., −1n.). This value is close to the actual markup of 12.04%. Qualitatively we do not observe differences between the static and the dynamic model. Markups estimated from the dynamic model are on average by about two percentage points higher than markups estimated from the static model. Again, the experiment shows that we can replicate our quasi-experimental results very well on average. Evaluating projects procured in the crisis in a pre-crisis environment (+1bl., −1n.), we predict average markups of 14.00% close to the actual markup of 13.96% depicted in column (3).

Projects before and during the crisis are not identical. The second experiment uses the pre-crisis projects and fixes the auction characteristics to answer the question: what happens to markups if the increased competition and lower backlog levels in the crisis would have been present before the crisis? We herewith control for changes in the composition of projects. We turn around the counterfactual from the previous experiment and apply it to pre-crisis projects (“−1bl.,+1n.”). Given one additional bidder and lower capacity utilization before the crisis on the very same projects before the crisis leads to counterfactual effects that support the evidence from the first counterfactual. Thus, our results are robust to a change in the type of projects in the crisis.

To model the government stimulus, we reduce the backlog of those bidders that won projects in the stimulus sector in the crisis. The question here is as follows: what is the quantitative effect of the government stimulus? We take the 17 stimulus projects that we were able to identify through parliament reports. We then randomly draw further projects in 2009 to reduce the backlog by the amount of the government stimulus directed to the construction sector, totalling 363.3 million Euro. The number of bidders increases by another one-third of a bidder relative to the crisis environment and the value of new contracts decreases by 2.5%. In this way, we aim to quantify the effects on bidding were there no government intervention. Lower backlogs and more bidders will lead to lower markups. To account for the fact that the government stimulus was implemented in the crisis, we apply the experiment to the crisis observations.

The results of the counterfactual analysis support our second hypothesis. If the government had not intervened, markups would have dropped by more than we observe. Our third experiment provides the evidence. Using the estimates from the static model, we observe a counterfactual markup of 10.02% for all projects, on average. Compared to the observed markup of 10.52%, this is an additional drop of half a percentage point. This drop is slightly larger when we consider the estimates from the dynamic model. This difference may illustrate the lost option of winning tomorrow.

Markups in columns (2) and (4) of Table 10 are for winning bids. While the markups are much higher for the winning bids, the general pattern, i.e. drop of the markup in the crisis, partial reversion of the drop in the markup due to the stimulus, is similar. The counterfactual analysis confirms the results we obtained for all bids, although the predictions for winning bids are less precise. Our results are robust to the specification of the model and we can also confirm our second hypothesis.

As in the pre–post quasi-experimental analysis, we also observe in our counterfactual analysis that markups are pro-cyclical. The additional demand due to the stimulus package increased markups. Moreover, possible crisis-related changes in the type of projects do not drive our results.

## 5 Discussion

Comparisons of pre-crisis and crisis markups consistently confirm Hypothesis 1: in the crisis, markups drop. Pre-crisis projects in the stimulus sector in general and the 17 stimulus projects in particular exhibit higher markups, but fall almost to the lower crisis markup level of projects not in the stimulus sector. It seems that the change in the competitive environment levels markups over different types of projects. We also provide evidence for Hypothesis 2, which says that the fiscal stimulus weakened the crisis drop in markups. Absent the stimulus, our counterfactual analysis shows that backlogs would have dropped further and (even) lower demand would have increased the number of bidders by more in the remaining auctions. Markups would have decreased by more, given the influence of backlog and number of bidders in the model. Thus, the two pieces of evidence consistently point to pro-cyclical markups.

While developing a new method to analyse a structural dynamic auction with unobserved heterogeneity and endogenous entry, Balat (2012) looks at a specialized construction subsector, namely road construction. These specialized construction firms do not face demand reduction from the private sector, because demand comes from the state. As the state increases spending on road construction as part of the stimulus, these firms actually experience increased demand. From the perspective of the economy-wide construction sector this is a special situation, because overall demand is reduced in the crisis, as we have shown. Balat (2012) finds that markups increase in the situation of demand increase, analogously to the markups decrease we find when demand decreases for the whole construction sector. Our results show that the pro-cyclicality of the markup is witnessed in both situations — increases and decreases of demand. Our results also show that the increased prices of government projects in Balat׳s (2012) study carry over to the other, more general construction sectors in our environment. The typical motive for government spending in a recession is stimulation of the economy. We find that the government also faces lower markups and therefore lower prices for its projects.

Domowitz et al. (1986) give empirical evidence on business cycles and their influence on the relationship between concentration and (average) price-cost-margins. They observe pro-cyclical margins in concentrated industries. As explanations, models of collusion and models of cost differences are suggested.^37^ With their industry-level data they do not give evidence allowing to discriminate between the explanations. Other recent studies by Nekarda and Ramey, 2010, Nekarda and Ramey, 2011 are also interested in testing the effects of government stimulus on markups — in particular in the context of different predictions from neo-classical and neo-Keynesian business cycle models. From neo-Keynesian models they expect countercyclical markups, given that prices are sticky while wages would increase due to demand increases, and therefore marginal cost would increase. The authors find pro-cyclical markups as we do, however, neither with economy-level nor with industry-level data do they find an effect of government stimulus on markups.

## 6 Conclusions

We estimate the cost distributions of construction firms based on detailed auction specific and firm level data. Based on the results, we measure the effects of competition on the bidding behavior and compare bidding behavior before and during the economic crisis. Our model permits quasi-experimental evidence on the effect of the crisis as well as the government׳s stimulus in the construction sector. The pro-competitive effect from free capacity and increased competition on the markup is estimated to be −1.5 percentage points overall and −3.3 percentage points for winning bids. Our evidence suggests that the fiscal stimulus, by injecting additional demand, counteracts the decline in markups. Thus, from two pieces of evidence we consistently find procyclical markups.

As our results are based on careful structural modeling and detailed data, both auction specific and firm specific, our results should not only contribute to research in industrial organization, but could also be a useful input for macroeconomics to model the effects of activist fiscal policy.

## Figures and Tables

**Table 1:** Variable descriptions.

Variable	Description
*Log* (*number of bidders*)	Log of number of bidders in the auction
*Backlog*	Backlog variable, standardized by firm mean and standard deviation
*Backlog sum*	Sum of backlog of the other bidders in the auction
*New orders*	Gross inflow of new contracts, countrywide, per month (mill. 2006 euros)
*Engineer estimate*	Engineer cost estimate for the construction project
*Log* (*employees*)	Log of the number of employees of a bidder
*KM*	Travel distance in kilometers from a bidder׳s location to the project site
*KM average*	Average of all other, competing bidders׳ distances between their location and the project site
*KM sum*	Sum of all other, competing bidders׳ distances between their location and the project site
*Same postal*	Bidder location has the same postal code as project site
*Same district*	Bidder resides in the district of the project site
*Same state*	Bidder resides in the state of the project site
*Heavy construction*	Dummy for auctions in heavy construction sector
*General contractor*	Dummy for bidders serving as “general contractors”
*Open format*	Dummy for auctions following the “open procedure”
*No. of potent. bidders*	Number of potential bidders

**Table 2:** Number of bidders, backlog and begin of the crisis.

Variable	(1)	(2)	(3)	(4)	(5)
*Panel A: Number of bidders*					
Constant	7.685	7.703	7.670	7.551	7.597
	(0.110)⁎⁎	(0.113)⁎⁎	(0.111)⁎⁎	(0.118)⁎⁎	(0.111)⁎⁎
Begin of crisis October 2008	1.263	1.107	1.602	1.386	1.650
	(0.174)⁎⁎	(0.265)⁎⁎	(0.320)⁎⁎	(0.178)⁎⁎	(0.193)⁎⁎
Placebo 1: August 2008		0.214			
		(0.273)			
Placebo 2: December 2008			−0.396		
			(0.313)		
Placebo 3: April 2008				−0.598	
				(0.195)⁎⁎	
Placebo 4: April 2009					−0.849
					(0.184)⁎⁎
Time trend	−0.001	−0.001	−0.001	−0.000	−0.001
	(0.000)⁎⁎	(0.000)⁎⁎	(0.000)⁎⁎	(0.000)	(0.000)⁎⁎

Observations	3974	3974	3974	3974	3974
Adjusted *R*-squared	0.014	0.014	0.015	0.017	0.020

*Panel B: Backlog*					
Constant	−0.450	−0.453	−0.450	−0.426	−0.439
	(0.010)⁎⁎	(0.010)⁎⁎	(0.010)⁎⁎	(0.011)⁎⁎	(0.010)⁎⁎
Begin of crisis October 2008	−0.257	−0.234	−0.265	−0.288	−0.296
	(0.016)⁎⁎	(0.025)⁎⁎	(0.028)⁎⁎	(0.017)⁎⁎	(0.018)⁎⁎
Placebo 1: August 2008		−0.032			
		(0.026)			
Placebo 2: December 2008			0.010		
			(0.028)		
Placebo 3: April 2008				0.118	
				(0.019)⁎⁎	
Placebo 2: April 2009					0.096
					(0.016)⁎⁎
Time trend	0.001	0.001	0.001	0.001	0.001
	(0.000)⁎⁎	(0.000)⁎⁎	(0.000)⁎⁎	(0.000)⁎⁎	(0.000)⁎⁎

Observations	29,842	29,842	29,842	29,842	29,842
Adjusted *R*-squared	0.105	0.105	0.105	0.106	0.106

*Notes*: Results from OLS estimations. In Panel A, the dependent variable is number of bidders in an auction; in Panel B, the dependent variable is bidders׳ backlog. Standard errors are in parentheses. ^⁎^ denotes 90% confidence level.

⁎⁎ Denotes 95 confidence level.

**Table 3:** Description of the sample.

Sample	Obs.
Auctions	3974
*minus* engineer estimate n.a.	2249
*minus* outliers	2069
*minus* missing data in variables of econometric model	2067

Firms	1655
*minus* bankrupt, dissolved	1566
*minus* non-matched	1342

*Notes*: The panel on the top shows the number of auctions in the sample and the reason why it is reduced in each line, together with the resulting new number of observations. Outliers are defined as auctions were either (1) even the smallest bid in an auction is much larger than the engineer estimate ($\min_{i}[{\mathrm{bid}}_{i}/\mathrm{Eng}.\;\mathrm{est}.]>1$⅔), or (2) even the largest bid is much smaller than the engineer estimate ($\min_{i}[\mathrm{Eng}.\;\mathrm{est}./{\mathrm{bid}}_{i}]>1$⅔). On the bottom panel one finds the number of firms that submitted bids. 1342 firms were matched to the external data providing additional firm level data. 96.0% of bids were made by these 1342 firms.

**Table 4:** Summary statistics.

Variable	2006		2007		2008		2009	
	Mean	S.D.	Mean	S.D.	Mean	S.D.	Mean	S.D.
*Firms*								
No. of firms	496		472		453		586	
Foreign	13		16		21		37	
Employees	133.1	466.6	196.5	706.4	148.1	490.4	174.5	616.7
Total assets (mill.)	15.61	79.54	26.15	166.53	19.77	89.05	19.35	83.65
								
*Auctions*								
Number of auctions	518		482		480		587	
Building	393		308		250		334	
Heavy	110		163		215		224	
Cost estimate (mill.)	1.89	3.06	2.33	4.77	2.65	5.83	2.81	9.65
Winning bid (mill.)	1.81	3.10	2.16	5.08	2.49	6.03	2.76	11.41
New orders (mill.)	1687	172.3	1778	194.9	1775	203.4	1684	170.6
*Bids*								
Number of bids	3553		3414		3404		4474	
Bid (mill.)	1.89	3.03	2.15	4.83	2.32	5.46	2.27	7.19
Distance (km)	123.83	121.85	123.89	121.06	138.69	121.33	134.73	121.93
Backlog	−0.306	0.641	−0.004	0.836	0.218	0.759	0.363	0.875
Same district	0.163	0.367	0.162	0.365	0.115	0.317	0.135	0.339
Same state	0.556	0.491	0.549	0.492	0.459	0.493	0.476	0.495
Same postal code	0.049	0.214	0.048	0.211	0.032	0.174	0.037	0.187
								
*Competition*								
Actual bidders	7.48	3.23	7.45	2.90	7.44	3.37	8.01	3.13
Potential bidders (def. 1)	29.00	10.34	28.94	10.57	27.77	10.79	31.83	13.32
Potential bidders (def. 2)	702.80	241.16	661.10	221.02	622.78	218.20	662.34	223.00

*Notes*: Monetary values in 2006 euros. “Total assets” and “Employees” neglect one very large foreign company which accounts for four bids in 2008 and 2009; inclusion unduly inflates the means.

**Table 5:** Descriptive statistics for engineer estimates, winning bids and second-ranked bids.

Number of bidders	(1)	(2)	(3)	(4)
	Engineer estimate	Winning bid	[(2)−(1)]/(1) (%)	Observations
2	3832	4020	4.9	35
3	6681	7411	10.9	100
4	2301	2147	−6.7	186
5	2812	2744	−2.4	251
6	3029	2947	−2.7	276
7	2387	2298	−3.7	254
8	2267	2043	−9.9	250
9	1861	1614	−13.3	216
10+	1361	1144	−15.9	499

Total	2432	2321	−4.6	2067


	*Money left on the table*			
Number of bidders	(5)	(6)	(7)	(8)
	All	Not stimulus sector	Stimulus sector	Stimulus project
2	16.1%	15.7%	17.5%	
3	10.9%	12.5%	6.6%	18.6%
4	10.9%	10.9%	11.0%	9.6%
5	8.4%	8.2%	9.2%	38.9%
6	8.8%	8.4%	10.1%	12.7%
7	6.9%	7.2%	5.4%	3.0%
8	6.4%	6.3%	6.9%	2.3%
9	6.2%	6.3%	5.8%	0.2%
10+	6.0%	6.0%	5.6%	9.8%

Total	7.7%	7.6%	8.0%	10.5%
Observations	2067	1733	317	17

*Notes*: Group “10+” contains all auctions with 10 or more bids. Engineer estimate and winning bid are in thousands of 2006 euros. Money left on the table, for each auction *j*, is $[{\mathrm{bid}}_{j}^{\left(2\right)}-{\mathrm{bid}}_{j}^{\left(1\right)}]/{\mathrm{bid}}_{j}^{\left(1\right)}$. The superscript indicates the order statistic of the bid.

**Table 6:** Determinants of the distribution of bids.

Variable	Scale, *λ*		Shape, ρ×1000	
	(1)	(2)	(3)	(4)
	Coefficient	Std. error	Coefficient	Std. error
Log (number of bids)	−16,334.38⁎⁎	3416.4	276.2⁎⁎	86.1
Backlog	2919.62⁎⁎	741.0	−39.6	20.7
Backlog sum	1219.44⁎⁎	236.1	31.9⁎⁎	5.5
New orders	23.70⁎⁎	2.8	0.2⁎	0.1
Engineer estimate	1.13⁎⁎	4.0E−03	5.0E−05⁎⁎	3.0E−06
Log (employees)	−58.71	351.4	139.7⁎⁎	10.3
KM	−6.68	7.2	−0.9⁎⁎	0.2
KM average	−87.95⁎⁎	22.0	−0.7	0.4
KM sum	38.72⁎⁎	4.2	0.1	0.1
Same postal	−8303.24⁎⁎	2566.4	−170.2⁎	75.0
Same district	43.73	1639.5	46.8	38.7
Same state	−5268.69⁎	2055.8	−168.6⁎⁎	46.7
Heavy construction	5986.82	3082.3	−69.8	40.2
General contractor	39,944.69⁎⁎	10,008.8	929.1⁎⁎	100.9
Open format	9614.52⁎⁎	1227.3	133.9⁎⁎	48.4
Number of potential bidders	−8.95	49.3	8.1⁎⁎	1.2
Constant	−12,684.13	9699.5	1533.1⁎⁎	243.5

Observations	14,845			

*Notes*: Column (1) shows the coefficients estimated for the scale parameter *λ* of the Weibull distribution of bids. Column (3) is for the shape parameter *ρ*. Standard errors are in the columns (2) and (4).

⁎ Significance at the 5% level.

⁎⁎ Significance at the 1% level.

**Table 7:** Markups for all projects, in the stimulus sector and for stimulus projects.

Outcome	All bids			Winning bids		
	(1)	(2)	(3)	(4)	(5)	(6)
	All projects	Stimulus sector	Stimulus projects	All projects	Stimulus sector	Stimulus projects
*Observed markups*						
Pre-crisis	12.04	14.91	NA	22.94	27.54	NA
	(0.17)	(0.51)		(0.70)	(1.87)	
Crisis	10.52	11.70	13.52	19.66	20.04	20.24
	(0.19)	(0.59)	(1.90)	(0.82)	(2.05)	(5.77)

Difference	−1.51	−3.21		−3.29	−7.50	
	(0.27)⁎⁎	(0.82)⁎⁎		(1.15)⁎⁎	(3.03)⁎	

*Notes*: Markups calculated for observed covariates, before and during the crisis. Crisis starts with bids submitted in October 2008. “Stimulus sector” refers to projects where the principal also conducts projects that were part of the government stimulus of 2009, but these projects are predominantly not part of the stimulus itself. “Stimulus projects” are projects in the stimulus sector (per definition), and were identified as being projects specifically added by the government in the stimulus program. Standard errors are in parentheses. ^⁎^ stands for significance at the 5% level.

⁎ Significance at the 5% level.

⁎⁎ Significance at the 1% level.

**Table 8:** Determinants of the distribution of bids before and during the crisis (R1).

Panel A: pre-crisis	Scale, *λ*		Shape, ρ×1000	
	(1)	(2)	(3)	(4)
	Coefficient	Std. error	Coefficient	Std. error
Log (number of bids)	−9986.13⁎	3966.07	196.75	151.33
Backlog	4897.38⁎⁎	1066.52	−53.66	29.64
Backlog sum	1808.35⁎⁎	343.46	18.30⁎	7.51
New orders	19.73⁎⁎	3.97	−0.11	0.11
Engineer estimate	1.16⁎⁎	4.84E−03	4.53E−05⁎⁎	4.50E−06
Log (employees)	154.30	386.74	149.52⁎⁎	13.28
KM	4.46	7.31	−0.83⁎⁎	0.27
KM average	−69.42⁎⁎	23.53	0.94	0.92
KM sum	31.73⁎⁎	4.87	0.14	0.13
Same postal	−4324.81	3439.67	−118.45	95.71
Same district	−2084.52	2351.82	−76.60	53.53
Same state	−1389.37	2141.90	−199.35⁎⁎	60.73
Heavy construction	−2829.70	3859.18	−204.69⁎⁎	54.54
General contractor	34,211.86⁎⁎	11,502.13	1227.49⁎⁎	127.83
Open format	3753.80⁎⁎	1444.84	283.53⁎⁎	61.33
Number of potential bidders	16.27	61.28	4.05⁎	1.93
Constant	−20,402.11	11,862.81	2020.85⁎⁎	398.36

Observations	9638			

Panel B: crisis	Scale, *λ*		Shape, ρ×1000	
	(1)	(2)	(3)	(4)
	Coefficient	Std. error	Coefficient	Std. error


Log (number of bids)	−33,491.87⁎⁎	8083.09	352.51⁎	170.22
Backlog	−902.68	1603.58	−1.71	32.25
Backlog sum	−3.70	500.65	23.40⁎	11.44
New orders	34.24⁎⁎	8.05	1.29⁎⁎	0.25
Engineer estimate	1.09⁎⁎	6.41E−03	3.93E−05⁎⁎	4.24E−06
Log (employees)	−1297.21	805.14	110.20⁎⁎	19.83
KM	−24.30	19.36	−0.50	0.35
KM average	−210.20⁎⁎	57.39	−2.31⁎⁎	0.68
KM sum	55.06⁎⁎	9.11	−0.03	0.13
Same postal	−10,378.43⁎	4468.64	−62.51	294.68
Same district	5958.60	3428.79	214.65	135.67
Same state	−17,592.44⁎⁎	4989.28	35.56	82.93
Heavy construction	15,747.73⁎	7060.78	91.62	89.80
General contractor	2149.23	18,139.25	523.34⁎⁎	177.79
Open format	22,672.04⁎⁎	2993.34	−228.75⁎	111.53
Number of potential bidders	−43.98	90.57	11.51⁎⁎	1.75
Constant	26,225.27	24,179.93	188.18	568.55

Observations	5207			

*Notes*: Column (1) shows the coefficients estimated for the scale parameter *λ* of the Weibull distribution of bids. Column (3) is for the shape parameter *ρ*. Standard errors are in the columns (2) and (4).

⁎ Significance at the 5% level.

⁎⁎ Significance at the 1% level.

**Table 9:** Markups for main and alternative specifications.

Panel A: data sample	Base model	R1	R2	R3	R4
		Sample split	Omit firm 1	No eng. est.	Outliers
*All bids*					
Pre-crisis	12.04	12.17	12.04	35.16	16.44
	(0.17)	(0.17)	(0.19)	(0.23)	(0.20)
Crisis	10.52	10.04	10.33	31.17	13.00
	(0.19)	(0.20)	(0.20)	(0.29)	(0.21)
Difference	−1.51	−2.14	−1.71	−3.99	−3.43
	(0.27)⁎⁎	(0.27)⁎⁎	(0.30)⁎⁎	(0.38)⁎⁎	(0.32)⁎⁎

*Winning bids*					
Pre-crisis	22.94	23.16	23.31	46.14	27.25
	(0.70)	(0.69)	(0.80)	(0.67)	(0.72)
Crisis	19.66	19.03	19.85	41.93	21.70
	(0.82)	(0.83)	(0.91)	(0.95)	(0.81)
Difference	−3.29	−4.12	−3.46	−4.21	−5.55
	(1.15)⁎⁎	(1.14)⁎⁎	(1.31)⁎⁎	(1.19)⁎⁎	(1.18)⁎⁎

Panel B: model specification		R5	R6	R7	R8
		FE 7 largest	Unobs. het.	End. entry	Dynamic model
*All bids*					
Pre-crisis		11.99	5.98	11.87	13.96
		(0.17)	(0.18)	(0.002)	(0.19)
Crisis		10.45	5.04	10.36	12.01
		(0.19)	(0.21)	(0.003)	(0.22)
Difference		−1.54	−0.93	−1.51	−1.95
		(0.27)⁎⁎	(0.29)⁎⁎	(0.008)⁎⁎	(0.31)⁎⁎

*Winning bids*					
Pre-crisis		22.97	14.54	25.72	25.17
		(0.70)	(0.77)	(0.006)	(0.74)
Crisis		19.58	12.42	23.15	21.21
		(0.82)	(0.98)	(0.007)	(0.86)
Difference		−3.39	−2.12	−2.56	−3.96
		(1.15)⁎⁎	(1.30)	(0.009)⁎⁎	(1.21)⁎⁎

*Notes*: Pre-crisis and crisis markup results for alternative models. Crisis starts with bids submitted in October 2008. R1: different cost distributions across periods. R2: firm supplying the engineer estimate omitted. R3: engineer estimate dropped. R4: outliers included. R5: fixed effects for seven largest firms. R6: unobserved heterogeneity model. R7: endogenous entry. R8: dynamic model. Standard errors are in parentheses. ^⁎^stands for significance at the 5% level.

⁎⁎ Significance at the 1% level.

**Table 10:** Counterfactuals for all bids and for winning bids.

Outcome	Base model		Dynamic model (R8)	
	(1)	(2)	(3)	(4)
	All bids	Winning bids	All bids	Winning bids
*Observed markups*				
Pre-crisis	12.04	22.94	13.96	25.17
	(0.17)	(0.70)	(0.19)	(0.74)
Crisis	10.52	19.66	12.01	21.21
	(0.19)	(0.82)	(0.22)	(0.86)
Difference	−1.51	−3.29	−1.95	−3.96
	(0.27)⁎⁎	(1.15)⁎⁎	(0.31)⁎⁎	(1.21)⁎⁎
*Crisis experiments*				
1: +1bl.,−1n.	12.53	21.30	14.00	22.84
	(0.19)	(0.79)	(0.22)	(0.83)
Difference	−2.01	−1.65	−1.99	−1.63
	(0.03)⁎⁎	(0.10)⁎⁎	(0.03)⁎⁎	(0.10)⁎⁎
2: −1bl.,+1n.	10.05	20.85	11.98	23.08
	(0.17)	(0.71)	(0.19)	(0.74)
Difference	−1.98	−2.09	−1.98	−2.09
	(0.05)⁎⁎	(0.23)⁎⁎	(0.05)⁎⁎	(0.23)⁎⁎
*Stimulus experiment*				
3:−363.3 mill Euro,+1/3n.	10.02	19.17	11.40	20.62
	(0.19)	(0.83)	(0.22)	(0.87)
Difference	−0.51	−0.48	−0.61	−0.59
	(0.01)⁎⁎	(0.03)⁎⁎	(0.01)⁎⁎	(0.04)⁎⁎

*Notes*: Markups calculated for observed covariates, before and during the crisis. Crisis starts with bids submitted in October 2008. The first experiment assumes that backlog is lower before the crisis by one standard deviation (“−1bl.”) and that an additional bidder is present in each auction (“+1n.”). The second experiment assumes that backlog is higher in the crisis by one standard deviation (“+1bl.”), mirroring the higher usage of capacity before the crisis; and that one bidder less is present in each auction (“−1n.”), to capture the lower level of competition taking place before the crisis. The third experiment manipulates the backlog of winning bidders in the crisis and the number of bidders (“+1/3n.”) to offset government stimulus worth 363.3 million Euro. Columns (1) and (3) show the results for all bids, (2) and (4) for winning bids only. Standard errors are in parentheses. ^⁎^ Significance at the 5% level.

⁎⁎ Significance at the 1% level.

## Appendix A Econometric models of main robustness checks

In this appendix, we explain how we implemented the dynamic model, the model with endogenous entry and the model with unobserved auction specific heterogeneity for structural estimation. All models are based on our main econometric model. As discussed in Section 3.2, we use the sealed-bid data to estimate the parameters of the basic model as a function of auction and bidder characteristics following the approach developed by Guerre et al. (2000).

### A.1 Dynamic model

Our main specification does not consider that firms may price into their bids also the lost option value of winning today versus winning later. Thus, we may underestimate markups in both periods and only obtain a lower bound for the estimated drop in markups in the crisis. We illustrate the econometric model of bidders׳ dynamic decision based on the estimated distribution of their construction cost following Jofre-Bonet and Pesendorfer (2003).^38^ They estimate a dynamic game in which bidders play sequential equilibria and use symmetric Markovian strategies, i.e., given the state variables bidders are symmetric and follow the same bidding strategy once they are in the same state.

Let Q=(X,Z′,N) with $Z\prime =Z\backslash (s_{i},s_{-i})$, where *s*_*i*_ is the backlog of bidder *i* and $s_{-i}$ the backlog of all other firms.^39^ We define the hazard function of bids submitted by bidder *i* as

(8) h^(·|Q,si,s−i)=g^(·|Q,si,s−i)1−G^(·|Q,si,s−i),

where G^ and g^ are the estimated distribution function and the estimated density function of our main specification. Using (8), the first order condition of optimal bids yields the following equations for privately known cost:

(9) c^i=ϕ(b|Q,si,s−i,h,V)=b−1∑j∈N\ih^(b|Q,sj,s−j)−β∑j∈N\ih^(b|Q,sj,s−j)∑l∈N\ih^(b|Q,sl,s−l)[Vi(ω(Q,s,i))−Vi(ω(Q,s,j))].

where *V* is the value function and *ω* the transition function. Eq. (9) provides an explicit expression of the privately known cost that involves the bid, the hazard function of bids and the value function. Once we know the value function and the transition function, we are able to back out cost from bids.

The transition function *ω* describes how the backlog evolves over time and is exactly defined as in Jofre-Bonet and Pesendorfer (2003). (See also Section 3.4.) The key idea to obtain an estimate for the value function *V* is to draw a random sample of states and numerically solve the explicit expression for the value function derived in Jofre-Bonet and Pesendorfer (2003) for every bidder on this grid and approximate it by a polynomial outside the grid. We draw 200 contracts randomly. For the quadratic polynomial, we run a robust regression including firm fixed effects. Our simulation sample includes 398 bidders. As a robustness check, we also run the static specification with this (reduced) sample and obtain markups that are very close to the original ones.

### A.2 Endogenous entry

In the data we observe that not all bidders participate in all auctions. This may indicate that bidders incur entry costs and ignoring them may yield biased markups. With the estimates of the distribution of bidders׳ costs at hand, we illustrate how we estimate bidders׳ entry cost based on observed entry behavior following Athey et al. (2011).

If participation is costly, then bidders behavior is modelled as a two stage decision process. First, each bidder incurs a cost *K*_*i*_ to gather information and enter the auction.^40^ Once the entry cost *K*_*i*_ is paid, bidder *i* learns her cost for the construction site, *c*_*i*_, and may bid in the auction. Actual bidders are denoted by the set N; bidders acquiring information are called participants, and we denote the set of participants by M,N⊆M with M=1,…,M.

Bidder *i*׳s ex ante expected profit from participating is

(10) $$\Pi_{i}(p)=\underset{N\subseteq M}{\sum }\pi_{i}(N)\mathrm{Pr}[N|i\;\mathrm{enters,}\;\mathrm{opponents}\;\mathrm{play}\;p_{-i}]$$

where $p=(p_{1},\ldots ,p_{N})$ is the profile of entry probabilities, and $\pi_{i}(N)$ is bidder *i*׳s profit in the auction when N bidders participate. Entering the auction is optimal, if the expected profit $\Pi_{i}(p)$ exceeds the entry cost *K*_*i*_. An asymmetric entry equilibrium ($p_{M}$) exists, but in general it need not be unique (Li and Zhang, 2014).

As before, let X denote the set of auction characteristics and Z denote the set of bidder characteristics. Based on estimated bidders׳ construction costs, we are able to calculate bidders׳ profits and then also their entry cost:

(11) $$\Pi_{i}(X,Z,N)=\underset{N\subseteq M}{\sum }\pi_{i}(X,Z,N,M)\mathrm{Pr}[N|X,Z,M,i\in N],$$

where Pr[N|X,Z,M,i∈N] is the probability that N bidders enter given that *i* enters. We use the sealed bid data to construct an estimate of bidder׳s beliefs about opponent entry. The distribution of bidder entry is binomial, as is the distribution of opponent entry. In particular,

(12) $$\mathrm{Pr}[N|X,Z,M,i\in N]=\underset{j\neq i\in N\subseteq M}{\prod }p_{j}(X,Z,N)\underset{j\in M\backslash N}{\prod }(1-p_{j}(X,Z,N)),$$

with the parametric specification of $p_{i}(X,Z,N)$ as

(13) $$p_{i}(X,Z,N)=\frac{\exp(\alpha_{X}X+\alpha_{Z}Z+N\alpha_{N})}{1+\exp(\alpha_{X}X+\alpha_{Z}Z+N\alpha_{N})},$$

The parameters of the model are estimated by maximum likelihood. Table 2 in the Online Appendix reports the results.

Eq. (12) shows that each possible combination of entrants from the auction-specific set of potential bidders has a different probability of being realized. The markups result then from entry simulations based on the logit results and trim the distribution of bid draws by choosing all draws of bids within +/−6 standard errors of the auction mean bid.

### A.3 Unobserved heterogeneity

For the model with unobserved heterogeneity, we follow Athey et al. (2011) and Krasnokutskaya (2011) and replace (5) by a mixture of the distributions where the bids are again Weibull distributed and the unobserved heterogeneity *u* is Gamma distributed. Conditional on the observable auction and firm characteristics (X,Z), and the set of bidders identities N, the joint distribution of bids in a given auction is the distribution $G_{i}(\cdot |X,Z,$ u,N) and equal to

(14) $$G_{i}(b_{i}|X,Z,u,N)=1-\exp\{-u{\left(\frac{b_{i}}{\lambda_{i}(X,Z,N)}\right)}^{\rho_{i}(X,Z,N)}\},$$

where $\lambda_{i}(X,Z,N)$ is the scale and $\rho_{i}(X,Z,N)$ is the shape of the Weibull distribution. Both are parameterized by linear functions of *λ* and *ρ* as before and *u* has zero mean and variance *θ*. We estimate the parameters of the model, (λ,ρ,θ), by maximum likelihood estimation.

## Appendix B Supplementary data

Application 1

Application 2

## Footnotes

1 Financial markets in 2006 did not foresee the collapse of subprime mortgage markets in early 2007 nor the fire sale of Bear Stearns in March 2008 to JPMorgan Chase to avoid bankruptcy, nor the collapse of Lehman Brothers on September 12, 2008, nor the bailout of American International Group (AIG), etc.

2 As we are mainly interested in the effect of the recent economic crisis on firms׳ markups, we do not account for endogenous entry. We estimate a model with entry cost as one of our robustness checks, though.

3 Nekarda and Ramey, 2010, Nekarda and Ramey, 2011 test the predictions of neo-classical and neo-Keynesian models aggregated on the level of industries and find pro-cyclical or acyclical markups. Ramey (2011) and Parker (2011) discuss the evaluation of stimulus packages.

4 For a survey on nonparametric identification and estimation of auction models see Athey and Haile (2005). For surveys of empirical studies of auctions see Hendricks and Paarsch (1995) or Laffont (1997).

5 Bajari and Hortaçsu (2005) provide evidence from experimental data that assesses the reasonableness of structural estimates.

6 In Jofre-Bonet and Pesendorfer (2003), firms price into their bids also the lost option value of winning today versus winning later. Our static model does not account for this additional effect: we attribute some of the lost option value to the (technical) cost estimate of the project and therefore underestimate markups in both periods. The option value is lower in the crisis and thus our main specification gives a lower bound for the estimated drop in markups in the crisis. In Athey et al. (2011), firms incur entry costs to gather information before participating in the auctions. Ignoring these costs may yield biased estimates for the markups.

7 For an introduction to auction models in general and first-price sealed bid auction models in particular, see Krishna (2009).

8 This is a commonly made assumption in the empirical auction literature. From personal conversations with firms we inferred that it is also suitable for our environment. Before submitting their bids, bidders in these auctions usually know who is potentially capable to complete the construction and is likely to bid.

9 In principle, we could model a secret (random) reserve price and estimate the distribution of sellers׳ valuation as in Li and Perrigne (2003). In our sample, we however do not observe any rejected bids to obtain an estimate for that distribution.

10 Note that — anticipating our empirical specification — the asymmetry across bidders in our paper comes from variation in observable firm characteristics such as distance to the construction site or number of employees.

11 The conditions for a unique equilibrium are provided in Maskin and Riley (2003) and Lebrun (2006).

12 If there were only one bidder, which we do not observe in our data, the equilibrium strategy would be different.

13 Assuming a multiplicative structure for the common shock and idiosyncratic private information, Krasnokutskaya (2011) derives sufficient conditions under which such a model is identified and proposes a nonparametric estimation procedure which results in uniformly consistent estimators of the distributions of the common shock and bidders׳ idiosyncratic costs. As a robustness check, we also estimate a model that accounts for auctions specific unobserved heterogeneity. The results are robust. For details, see Section 4.3 and A.3.

14 In the empirical analysis, our N also includes a measure for potential bidders. For details see Section 3.4. Herewith, we account for differences between potential and participating bidders, although bidders׳ entry decisions are for simplicity not formalized in the theoretical model that describes our main specification.

15 If submitting a bid in this type of auctions is costly and bidders observe their project completion cost before having to pay the participation cost as in Samuelson (1986), then the estimation of the bid distribution could be problematic. Even if bid submission is costly, we argue based on our conversation with Josef Aicher, a university expert in procurement law, that bidders do not observe their project completion cost before they pay participation cost as projects are far too complex to know that. As stated above, we also control for endogenous entry as a robustness check and find our results to be robust.

16 Actually, Li and Zhang (2014) show identification in the affiliated value model with endogenous entry. For a general discussion on identification in first-price auctions, see Athey and Haile (2005).

17 Note that in our case *G*_*i*_ also depends on firm characteristics, Z. Thus, we have to calculate *G*_*j*_ for all bidders *j* in Eq. (6). This is different to other applications such as Athey et al. (2011) and Krasnokutskaya (2011). In these applications there are at most two groups of bidders and *G*_*j*_ is equal among these groups.

18 As the reserve price in Austrian procurement auctions is secret, we do not need to account for it in the estimations. Note that we observe all bids, thus there is also no selection in the data.

19 We show trailing moving averages over 12 months to smooth out seasonal variation.

20 Backlog is calculated as in Jofre-Bonet and Pesendorfer (2003). When a firm wins a project, it is added to the backlog and then linearly worked off over the construction period. For the exact definition of our backlog measure see Section 3.4.

21 For example, Somaini (2011) and Hong and Shum (2002) provide evidence of interdependent valuations in the construction industry.

22 Even if the quantity of a component changes later in the process of construction, as due to unexpected difficulties, for example, more cement is required, this is included in the offer. Only the price for cement cannot change.

23 Cantillon (2008) shows that the composition of strong versus weak bidders is the relevant factor.

24 Li and Zheng (2009) show that it is possible that as the number of competitors increases bidding may become less aggressive.

25 We thank an anonymous referee for pointing that out to us.

26 See Campo et al. (2011) for a semi-parametric approach to estimate risk-aversion.

27 Engineers of the firm that provided the data calculate the engineer estimate. It is a technical cost estimate that this firm uses for preparing its bids. To our knowledge the missing values are random and there is no specific reason why we do not observe them. However, we run various robustness checks to obtain a better assessment. See Section 4.3 for details.

28 We defined a cutoff for outliers when for an auctions either (1) even the smallest bid in an auction is much larger than the engineer estimate ($\min_{i}[{\mathrm{bid}}_{i}\backslash \mathrm{Eng}.\;\mathrm{est}.]>1$⅔), or (2) even the largest bid is much smaller than the engineer estimate ($\min_{i}[\mathrm{Eng}.\;\mathrm{est}.\backslash {\mathrm{bid}}_{i}]>1$⅔).

29 If a firm has multiple plants we still can only calculate the distance between the headquarter and the construction site as we do not have any information on plants. Only for larger firms we know their subsidiaries and can calculate the distances accordingly.

30 If we compare the mean distance of the winning bidder over time, we also observe that winners actually drive longer distances in the crisis.

31 We estimate the bid distribution with the bids where all covariates are available (*n*=14,845). For the calculation of markups, neglecting missing values would introduce systematic error in Eq. (6). To avoid this error, we impute, for example, a missing value for the number of employees by the median number of employees of all other firms except for the largest seven bidders in the sample. Adding the bids with one or more imputed covariates yields *n*=15,738, the actual number of bids submitted in the 2067 auctions. In the subsequent investigations into the calculated markups, we revert to exclude markups from bids with imputed covariates.

32 The seven largest firms are defined by how often they participated over the four years we observe our data. We used 500 participations as the cutoff value and then created a firm fixed effect for each of these firms.

33 For details of the empirical implementation of the last three models see Appendix A. Estimation results for the bid distribution of the robustness checks are shown in the Online Appendix.

34 See Table 1 in the appendix for the underlying estimation results.

35 Without reporting them, our results are also robust to the inclusion of firm dummies for the ten largest firms. These firms have participated at least 400 times in our data.

36 The results for the cost functions (backed out of the base model and the dynamic model) are available upon request from the authors.

37 Cyclical markups have also received attention in the context of collusion, especially after Rotemberg and Saloner (1986). Rotemberg and Saloner predict countercyclical markups from their model. Some of the influential literature that followed favors procyclical markups as a consequence of collusive incentives (Haltiwanger and Harrington, 1991, Staiger and Wolak, 1992; also see Bagwell and Staiger, 1997, and Fabra, 2006, for models with both pro- and countercyclical outcomes possible).
