# Supplementary material for: Competition in the economic crisis: Analysis of procurement auctions
Source: Eur Econ Rev. 2015 Jan;73:35–57. doi: 10.1016/j.euroecorev.2014.10.007 (PMC4318169; doi:10.1016/j.euroecorev.2014.10.007)
Supplement: Application 1 [file mmc1.pdf]

Online Appendix for "Competition in the economic crisis: Analysis of procurement auctions"

Klaus Gugler* Michael Weichselbaumer† Christine Zulehner‡

October 2014

*Vienna University of Economics and Business, Address: Welthandelsplatz 1, A-1020 Vienna, Austria, Email: klaus.gugler@wu.ac.at.

†Vienna University of Economics and Business, Address: Welthandelsplatz 1, A-1020 Vienna, Austria, Email: michael.weichselbaumer@wu.ac.at.

‡Goethe University Frankfurt, Austrian Institute of Economic Research Vienna and CEPR, Address: Grueneburgplatz 1, D-60323 Frankfurt am Main, Germany, Email: zulehner@safe.uni-frankfurt.de.

Table 1: Alternative estimations of the determinants of bidders' valuations

	R2<br>Omit firm 1	R3<br>No eng. est.	R4<br>Outliers	R5<br>Fixed eff. largest 7	R6<br>Unobs. het.
	λ	λ	λ	λ	λ
Log(number of bids)	-29,633.33**	-27,462.09	-28,485.37**	-16,097.69**	19.01
	4,930.81	37,056.91	5,950.01	3,382.39	3,061.28
Backlog	1,551.56	-2,838.99	2,211.97	2,106.20**	-65.69
	868.21	7,545.58	1,180.40	781.82	389.88
Backlog sum	1,138.61**	-559.35	-495.08	975.88**	381.21
	257.55	2,246.73	425.66	250.79	300.02
New contracts	25.29**	-0.96	64.01**	22.36**	2.02
	3.26	42.26	5.23	2.97	4.60
Engineer estimate	1.13**		1.14**	1.13**	0.90**
	4.09E-03		4.83E-03	3.80E-03	4.56E-3
Log(employees)	815.43	51,798.26**	1,788.17**	2,846.66**	-42.56
	441.26	3,726.67	593.30	509.95	127.44
KM	24.49*	-555.48**	-43.17**	1.44	3.93
	10.07	131.09	12.07	7.72	3.84
KM average	-162.22**	7,182.86**	-61.11	-92.20**	35.49**
	31.39	322.60	43.41	22.08	13.25
KM sum	50.99**	-134.26**	28.31**	36.11**	0.87
	5.50	35.87	6.28	4.18	3.34
Same postal	-7,527.49**	-98,939.45**	-23,187.41**	-10,325.05**	-2,294.83**
	2,588.06	31,484.86	3,469.82	2,626.85	1,011.08
Same district	1,477.66	-3,000.42	11,266.91**	1,130.19	423.28
	1,824.41	18,572.52	2,497.76	1,847.78	724.23
Same state	-9.19	-235,207.47**	-14,843.06**	-7,685.32**	-2,877.55**
	2,271.98	28,200.79	2,994.74	2,079.06	971.73
Heavy construction	9,601.49**	296,378.86**	58,129.87**	5,134.68	4,297.76
	3,426.96	23,607.66	4,579.40	3,189.35	3,518.31
General contractor	5,025,295.08**	2,759,590.32**	147,362.12**	39,814.25**	41,077.84**
	11,545.86	181,154.00	14,943.72	10,128.55	11,219.04
Open format	14,454.91**	240,937.03**	32,016.29**	9,917.39**	4,403.20**
	1,501.05	19,396.39	1,941.63	1,259.69	1,439.22
Number of potential bidders	-29.72	-4,212.01**	-392.67**	2.20	67.18
	56.05	601.20	86.96	49.86	70.26
Constant	-1,572.95	388,185.94**	-28,260.96	-17,819.32	-9,338.56
	12,734.78	120,161.92	17,145.14	10,067.51	11,340.71

Table 1: Alternative estimations of the determinants of bidders' valuations (continued)

	R2<br>Omit firm 1	R3<br>No eng. est.	R4<br>Outliers	R5<br>Fixed eff. largest 7	R6<br>Unobs. het.
	$\rho \times 1,000$	$\rho \times 1,000$	$\rho \times 1,000$	$\rho \times 1,000$	$\rho \times 1,000$
Log(number of bids)	283.86**	324.24**	973.74**	246.63**	0.41
	96.96	20.94	51.70	88.25	0.28
Backlog	-40.26	-4.56	-3.04	-43.48*	-2.67E-02
	22.45	3.16	10.03	21.35	3.62E-02
Backlog sum	34.19**	-6.82**	-11.51**	31.70**	-2.21E-02
	5.81	0.74	2.20	5.59	1.48E-02
New contracts	0.24*	3.36E-02**	0.42**	0.21*	
	9.27E-02	1.14E-02	4.27E-02	8.65E-02	
Engineer estimate	4.89E-05**		8.61E-05**	4.84E-05**	
	3.16E-06		2.55E-06	3.09E-06	
Log(employees)	151.03**	-7.86**	23.84**	115.90**	0.34**
	12.28	1.35	5.52	14.35	1.90E-02
KM	-0.84**	-0.11**	-0.22*	-0.96**	-1.38E-03**
	0.23	2.82E-02	9.28E-02	0.21	3.79E-E04
KM average	-1.09*	-0.66**	-0.67*	-0.76*	7.20E-03**
	0.43	0.11	0.28	0.38	1.76E-03
KM sum	0.12	6.31E-02**	8.76E-02	0.11	-8.28E-04**
	7.83E-02	2.11E-02	4.81E-02	7.11E-02	2.36E-04
Same postal	-155.75*	-161.46**	3.26	-174.03*	-0.38*
	78.34	21.07	48.88	75.44	0.17
Same district	55.42	103.77**	61.66	23.54	0.49**
	40.12	13.04	34.19	39.46	0.10
Same state	-173.09**	2.64	28.16	-124.07*	5.81E-02
	50.86	8.12	21.69	49.10	9.36E-02
Heavy construction	-95.56*	190.10**	-84.47**	-100.83*	-0.47**
	42.97	7.59	24.31	40.84	0.12
General contractor	935.26**	123.14**	647.64**	901.94**	3.39**
	113.17	19.27	46.54	101.69	0.33
Open format	113.73*	9.17	208.01**	140.65**	-0.15
	51.68	12.68	24.45	49.20	0.16
Number of potential bidders	8.21**	2.73**	9.19**	7.88**	-4.66E-03
	1.28	0.31	0.94	1.22	4.72E-03
Constant	1,484.10**	112.99*	-904.74**	1,682.88**	5.54**
	271.31	44.93	116.14	251.62	0.63
θ					2.49**
					7.66E-02
Fixed eff. 7 largest				Yes	
Observations	12,998	27,029	15,936	14,845	14,781

Notes: Alternative estimation results for Table 9 in the paper. R1 shows the coefficients for the Weibull model after the firm supplying the engineer estimate is omitted from the bid determinants estimation. Version R2 dropped the engineer estimate variable. R3 keeps "outliers"; outliers are defined as auctions where the smallest bid was much larger than the engineer estimate or the largest bid was much smaller than the engineer estimate; precisely: $\min_i[\text{bid}_i/\text{Eng. est.}] > 1\,^2/_3$, or $\min_i[\text{Eng. est./bid}_i] > 1\,^2/_3$. R4 included fixed effects for the seven largest firms, measured by the count of auction participations. Column R5 is derived from the unobserved heterogeneity model (two bids were dropped to achieve convergence). R7 and R8 have the same bid distribution model as the main model. $*$ ($**$) stands for significance at the 5% (1%) level. z-values in parentheses are below the coefficients.

Table 2: Determinants of entry before and during the crisis

	Pre-crisis		Crisis	
	(1)	(2)	(3)	(4)
	Coefficient	Std. Error	Coefficient	Std. Error
Backlog	-0.08**	0.017	0.22**	0.019
New orders	-0.32**	0.063	-0.31**	0.099
Engineer estimate	-0.02**	0.003	-0.02**	0.003
Log(employees)	0.85**	0.010	0.85**	0.013
KM	-0.61**	0.014	-0.62**	0.019
Same postal	-0.82**	0.142	-1.18**	0.245
Same district	1.32**	0.036	1.27**	0.051
Same state	1.58**	0.036	1.39**	0.047
Heavy construction	0.06*	0.026	-0.09*	0.036
General contractor	-0.56**	0.049	-0.50**	0.073
Open format	0.26**	0.035	0.28**	0.046
Major firm	3.09**	0.040	2.93**	0.054
Constant	-8.42**	0.126	-8.19**	0.189
Observations	1,731,001		839,360	
Pseudo R^2	0.421		0.410	

Notes: "Pre-crisis" shows the logit estimates of the model for entry before the crisis, "Crisis" for the crisis-period. $*$ ($**$) stands for significance at the 5% (1%) level. Standard errors are in the columns (2) and (4).
