# Supplementary material for: Competition in the economic crisis: Analysis of procurement auctions
Source: Eur Econ Rev. 2015 Jan;73:35–57. doi: 10.1016/j.euroecorev.2014.10.007 (PMC4318169; doi:10.1016/j.euroecorev.2014.10.007)
Supplement: Application 2 [file mmc2.zip › read_me.pdf]

Read-Me file for:
Gugler, Klaus, Michael Weichselbaumer and Christine Zulehner, "Competition in the economic crisis: Analysis of procurement auctions".

The Stata-do-files contain the code that generates the Figures and output for the tables in the article. Our data are proprietary. Researchers who want to replicate the results should contact Michael Weichselbaumer to organize local access at the Vienna University of Economics and Business.

Datasets

The following files are used in the do-files, in a subfolder named "files":

- data.dta
    - Main data set containing bids and covariates.
- data_figure2.dta
    - Data on monthly basis for Figure 2.
- data_figure3.dta
    - Data on monthly basis for Figure 3.
- entry.dta
    - Dataset for R7 (entry model), containing bid dummy and covariates for all combinations of auctions and bidders.
- contract200.dta
    - Result of random draw of 200 contracts for R8 (dynamic model).
- state50.dta
    - Result of random draw of 50 backlog states for R8 (dynamic model).

Programs

The Stata-do-files below generate the output for the Tables in the article and the Figures:

- figures_1_2_3.do
    - Figure 1
    - Figure 2
    - Figure 3
- descriptives.do
    - Dummy variables regression with Placebo dummies for the crisis start timing; summary statistics table by year; descriptive statistics for winning bids, engineer estimate and money left on the table.
    - Table 2
    - Table 4
    - Table 5
- bid_distribution.do
    - Estimates the Weibull bid distribution, which is the basis for the main results.
    - Markup calculation follows after the estimation of the bid distribution.
    - Counterfactuals 1, 2 and 3 are calculated afterwards.
    - Figure 4
    - Figure 5
    - Figure 6
    - Figure 7
    - Table 6
    - Table 7
    - Table 9
    - Table 10: Column (1) and (2)
- robustness_R1.do

    - Sample split, to obtain the results before and in the crisis with separate Weibull distribution estimates for each of the two periods.
    - Table 8
    - Table 9: Column R1
- robustness_R2.do
    - Drops the firm that provided the data.
    - Table 9: Column R2
- robustness_R3.do
    - Drops the engineer estimate from the explanatory variables.
    - Table 9: Column R3
- robustness_R4.do
    - Outliers included in the estimation of the bid distribution.
    - Table 9: Column R4
- robustness_R5.do
    - Fixed effects for the seven firms which bid most often.
    - Table 9: Column R5
- robustness_R6.do
    - Unobserved heterogeneity model, added as Gamma distributed auction heterogeneity to the Weibull distribution.
    - Table 9: Column R6
- robustness_R7.do
    - Entry model, which first estimates a Logit for bidding in the auction and then, according to the Logit results, makes random draws of entrants for each auction to calculate markups with estimated entry.
    - Table 9: Column R7
- robustness_R8.do
    - Dynamic model according to Jofre-Bonet and Pesendorfer (2003).
    - Table 9: Column R8
    - Table 10: Column (3) and (4)
